# The association of prokaryotic antiviral systems and symbiotic phage communities in drinking water microbiomes

**DOI:** 10.1038/s43705-023-00249-1

**Published:** 2023-05-04

**Authors:** Dan Huang, Mengting Maggie Yuan, Juhong Chen, Xiaoxuan Zheng, Dongsheng Wong, Pedro J. J. Alvarez, Pingfeng Yu

**Affiliations:** 1 College of Environmental and Resource Sciences, Zhejiang University, Hangzhou, China; 2 Department of Environmental Science, Policy, and Management, University of California, Berkeley, CA USA; 3 Department of Biological Systems Engineering, Virginia Tech, Blacksburg, VA USA; 4 Department of Civil and Environmental Engineering, Rice University, Houston, TX USA

**Keywords:** Microbial ecology, Water microbiology, Community ecology

## Abstract

Prokaryotic antiviral systems are important mediators for prokaryote-phage interactions, which have significant implications for the survival of prokaryotic community. However, the prokaryotic antiviral systems under environmental stress are poorly understood, limiting the understanding of microbial adaptability. Here, we systematically investigated the profile of the prokaryotic antiviral systems at the community level and prokaryote-phage interactions in the drinking water microbiome. Chlorine disinfectant was revealed as the main ecological driver for the difference in prokaryotic antiviral systems and prokaryote-phage interactions. Specifically, the prokaryotic antiviral systems in the microbiome exhibited a higher abundance, broader antiviral spectrum, and lower metabolic burden under disinfectant stress. Moreover, significant positive correlations were observed between phage lysogenicity and enrichment of antiviral systems (e.g., Type IIG and IV restriction-modification (RM) systems, and Type II CRISPR-Cas system) in the presence of disinfection, indicating these antiviral systems might be more compatible with lysogenic phages and prophages. Accordingly, there was a stronger prokaryote-phage symbiosis in disinfected microbiome, and the symbiotic phages carried more auxiliary metabolic genes (AMGs) related to prokaryotic adaptability as well as antiviral systems, which might further enhance prokaryote survival in drinking water distribution systems. Overall, this study demonstrates that the prokaryotic antiviral systems had a close association with their symbiotic phages, which provides novel insights into prokaryote-phage interactions and microbial environmental adaptation.

## Introduction

Microbial interactions are critical to microbiome composition and functions, which have broad ecological implications and important biotechnological applications [1, 2]. Recent developments in metagenomics have advanced our understanding of microbial interactions under environmental stress and shed light on the ecological drivers of microbial interactions [3–5]. Phages, known as prokaryotic viruses, are an important component of microbial assemblies [6]. Phages can affect microbial community through multiple patterns (e.g., cell lysis and horizontal gene transfer) [7]. However, our knowledge about prokaryote-phage interaction remains limited despite the increasing recognition of its prevalence and importance in microbial stability and functions. Therefore, it is necessary to elucidate factors that mediate prokaryote-phage interactions.

Prokaryotes have evolved various defense systems to prevent phage infection and prophage activation [8]. As an important mediator for prokaryote-phage interactions, antiviral systems can improve prokaryotic resistance to phage infection and thus prevent the rapid propagation of lytic phages [9, 10]. However, excessive defense may also mitigate beneficial horizontal gene transfer [11] and increase the prokaryotic metabolic burden in hostile environments [10]. Recent virome studies reveal that phages under environmental stress (e.g., heavy metals, pesticides) exhibit potential benefits for the prokaryotic community by compensating for auxiliary metabolic genes (AMGs) associated with pollutant degradation or detoxification [12, 13]. It remains largely unexplored how prokaryotic antiviral systems respond to environmental stress and their implications for prokaryote-phage interactions, which represents a critical knowledge for microbial adaptation to hostile environments.

Drinking water microbiome is important for water safety and public health. Nutrient limitation and residual disinfectants can inhibit microbial growth and shape the prokaryotic and phage community in drinking water distribution systems (DWDS) [14–16]. However, it remains elusive that prokaryotic antiviral systems and their association with prokaryote-phage interactions in DWDS. Addressing this critical knowledge gap will not only reveal the contribution of prokaryotic antiviral systems to prokaryote-phage interactions but also help understand how prokaryotes cope with hostile conditions like DWDS.

In this study, publicly available metagenomic datasets from the non-disinfected Netherlands DWDS and the disinfected UK DWDS were collected to explore prokaryotic antiviral systems and prokaryote-phage interactions [15]. The prokaryotic antiviral systems were comprehensively explored including the abundance, composition and prokaryotic carriers. Moreover, prokaryote-phage interactions in DWDS microbiomes were characterized in terms of phage and prokaryotic lysogenicity, as well as prokaryote-phage linkages. Additionally, antiviral systems and AMGs carried by phages were mined to understand the potential implications of the symbiotic phages. Mental tests were used to define the main environmental drivers of prokaryotic antiviral systems, prokaryote-phage interactions, and the potential benefits of phages.

## Methods

### Metagenomic sequence data collection and processing

All metagenomic sequence data of the DWDS microbiomes in this study were collected from NCBI and affiliated with bioproject number PRJNA533545 [15]. Specifically, 147 water samples were collected from 5 non-disinfection drinking water systems in the Netherlands (84 samples) and 7 chlorinated drinking water systems in the UK (63 samples), and samples were collected at two to four sites per DWDS, and the details of sampling location were provided in the Table S1. Additionally, the concentration of residual chlorine and other water quality parameters were described in Table S1 of Dai et al.’s article [15]. There was no significant difference in other measured water quality parameters except for phosphorus and residual chlorine [15], and the higher phosphate levels may reflect its use for corrosion control [17].

Sample treatment and DNA sequencing were described by Dai et al. [15], with each sample filtered through a 0.22 um filter with 15 L of water, followed by extraction of total DNA to construct sequencing libraries. All the metagenomic sequencing was performed on the Illumina Hiseq platform (2 × 250-bp read length). FastQC (v0.11.9) and Trimmomatic (v0.39) were used for the quality control of metagenomic raw reads with the default parameters [18, 19]. High-quality reads originating from each drinking water system were used for co-assembly by MEGAHIT (v1.2.8) [20] with kmer-min 35, kmer-max 115, k-mer-step 10, and min-contig-len 500, which resulted in 12 assembly files, including seven from the UK and five from the Netherlands systems. Then assembly quality was assessed with QUAST v5.0.2. (Table S2) [21].

### Identification, dereplication, and taxonomic classification of viral genomes

Putative viral contigs (length > 1 kb) were identified according to the SOP used in Sulivan Lab for viral identification based on VirSorter2 (v2.2.1) [22] and CheckV (v0.9.0) [23]. Firstly, VirSorter2 was run at a loose cutoff of 0.5 for maximal sensitivity, requiring hallmark gene on short seqs. Then CheckV was used to quality control the VirSorter2 results and also to trim potential host regions with default parameters. Then, the contigs in the output file “viruses.fna” were again determined by VirSorter2, and the resulting contigs were considered as free phages. Moreover, prophage integrated on prokaryotic genomes were identified by CheckV from all assembled contigs directly, and these contigs in the output file “proviruses.fna” were also identified by VirSorter2. Then the resulting contigs were identified as prophages. Finally, all viral contigs were dereplicated by CD-HIT (v4.8.1) with the parameters “-aS 0.8 -c 0.95” [24]. A total of 13,819 free viral contigs and 405 prophage contigs were identified, and N50 were 5913 and 19,018 bp, respectively (Table S3). DeePhage was used to make the judgment whether the phage was lytic or lysogenic (i.e., virulent or temperate) [25]. It is necessary to acknowledge that the sample processing protocol used by the original paper may result in the loss of some free phages. However, the main conclusion should not be undermined due to the identical protocol for sample collection and processing.

The open reading frames (ORFs) in viral contigs were predicted by Prodigal (v2.6.3) [26], and protein-coding amino acid sequences of ORFs were translated. Subsequently, the database of phylogenetically informative profile HMMs (ViPhOG v1, ftp://ftp.ebi.ac.uk/pub/databases/metagenomics/viral-pipeline/hmmer_databases) constructed by Luis et al. was used for viral taxonomic assignment of contigs in our study, where each model was specific to one viral taxon [27]. Specifically, query each viral protein sequence against the ViPhOG database with a per-domain independent *E*-value threshold of 0.00001. The resulting hits were further analyzed to predict the most likely and specific taxon for the whole contig according to the following criteria: (i) at least 20% of the genes matched the ViPhOG database, or if the contig had less than ten total genes, there should be at least two genes with hits; and (ii) at least 60% of these genes matched to the ViPhOG database were attributed to the same viral taxonomic unit.

### Taxonomic and functional analysis of prokaryotic genomes

After extracting the viral contigs, CD-HIT (v4.8.1) was used to dereplicate the remaining contigs (>500 bp) with the parameters “-aS 0.8 -c 0.95” [24]. Then, the ORFs in those contigs were predicted by Prodigal (v2.6.3) [26]. Subsequently, taxonomic classification at the species level for those contigs was performed based on the NCBI-NR database [28] via Diamond with *E*-value 1e-5 [29].

### Phage–host linkage analysis

In this study, the “in situ” and “ex situ” hosts for viral contigs were identified via two separate pipelines. For “in situ”, multiple methods were used to establish the link between viral contigs and the prokaryotic genome, including tRNA matching, CRISPR matching, and prophage localization on the prokaryotic genome. Specifically, (i) CRISPR spacer matching: CRISPR spacers were found on the sequences of the repeats using a machine learning approach by CRISPRCasTyper (--prodigal meta) [30], then SpacePHARER (CRISPR Spacer Phage-Host Pair Finder) was used as the tool for de novo prediction of phage-host relationships by comparing spacers and viral contigs at the protein level [31]. (ii) tRNA matching: the tRNA genes of viral contigs and local prokaryotic genome were identified by tRNAscan-SE (v2.0.9) [32] with parameters “-A” and “-B”, then the blastdb of prokaryotic tRNA genes was made, and the matching of viral and prokaryotic tRNA genes was executed using BLASTn with *E*-value 1e-10. Finally, perfect matches were selected for putative hosts. (iii) Host prediction of prophages: the prokaryotic genome in which the CheckV-identified prophage integrated was identified as the host for the corresponding prophage.

For “ex situ”, the putative phage hosts were predicted based on public databases IMG/VR v3 with *E*-value 1e-10 via (i) sequence similarity between viral contigs and a putative host genome, and (ii) matching viral contigs and CRISPR spacers in databases [13]. In our study, the analysis results from “ex situ” pipeline were only used to explore the polyvalent phages ratio changes together with “in situ” pipeline. Putative hosts with genus-level taxonomic annotations were retained for subsequent analysis, including the population distribution of hosts and the host range of linked phages [13].

### Identification of prokaryotic antiviral systems

DefenseFinder developed by Florian et al. in 2022 was a software that facilitates large-scale genomic analysis of antiviral defense systems [9]. In total, 60 antiviral families (151 subtypes of systems) were contained in DefenseFinder. In our study, the systematic and quantitative analysis of the antiviral arsenal of prokaryotes in DWDS was performed using DefenseFinder via an online service (https://defense-finder.mdmparis-lab.com/) with inputting prokaryotic and viral assembly files (fasta). Subsequently, the taxonomic composition of prokaryotic contigs carrying antiviral systems was further analyzed according to the previous taxonomic annotation of prokaryotic contigs. Moreover, CRISPRCasFinder was used to identify both CRISPR arrays and Cas proteins [33]. CRISPR spacers were extracted and counted by CRISPRCasTyper [30]. To avoid the potential errors caused by the difference in lengths of contigs with CRISPR-Cas, we normalized the number of CRISPR spacers with the contig length using the formula below:

$${{{{{{{\mathrm{M}}}}}}}} = \sum \left( {{{{{{{{\mathrm{N}}}}}}}}/{{{{{{{\mathrm{L}}}}}}}} \ast {{{{{{{\mathrm{A}}}}}}}}} \right)$$

N: the number of CRISPR spacers in contig

L: length of contig with CRISPR-Cas system (bp)

A: relative abundance of contig (tpm).

### Phage AMG identification and the structure prediction of AMG encoded proteins

DRAM-v in DRAM (v1.2.0) [34] and VIBRANT (v1.2.0) [35] were used to predict potential AMGs in viral contigs (including free viral contigs and prophages) together. Specifically, the “affi-contigs.tab” files of viral contigs generated by VirSorter2 were run through DRAM-v, and these AMGs with auxiliary scores 1, 2, and 3 were chosen, finally a total of 228 AMGs were identified by DRAM-v. In addition, VIBRANT was run with 4 ORFs per scaffold to constrain input sequences, and 805 AMGs were identified in this way. 62 AMGs were identified by both assays. Then, all AMGs have been annotated based on Kofam, PFAM, CAZy, NCBI Viral RefSeq, and VOGDB database. The structure of AMG encoded proteins was predicted by phyre2 in normal modeling mode (http://www.sbg.bio.ic.ac.uk/phyre2).

### Quantification and statistical analysis

In order to calculate the relative abundance of viral contigs and prokaryotic contigs, we mapped all non-redundant metagenomic reads against all viral and prokaryotic contigs here generated using BWA-MEM (v0.7.17) [36]. To reduce imprecise matching of reads, the reserved reads for contigs must meet the criteria that the mapping length of reads is more than 30 bp (bwa mem -k 30). The transcripts per kilobase million (TPM) values of contigs were determined by Python (v3.9). In addition, the relative abundance of phages and prokaryotes, AMGs, and antiviral systems were calculated by Python (v3.9) too. R (v4.2.0) was used to perform the alpha and beta diversity (PCoA based on the Bray Curtis distance between samples) analysis of prokaryotic (based on taxonomic annotation) and viral community structure profiles (based on sequence dissimilarity) via package “vegan” and “ggplot2” [37, 38]. The data normalization, Pearson correlations, Mantel test and all different tests were performed in Python (v3.9), and a *p* < 0.05 was considered statistically significant. Moreover, all figures in our study were drawn in R (v4.2.0), Cytoscape (v3.9.1) [23], and online plotting tools, including hiplot (https://hiplot-academic.com/) and chiplot (https://www.chiplot.online/).

## Results

### The abundance and composition of prokaryotes carrying antiviral system in DWDS microbiome

**Fig. 1:**
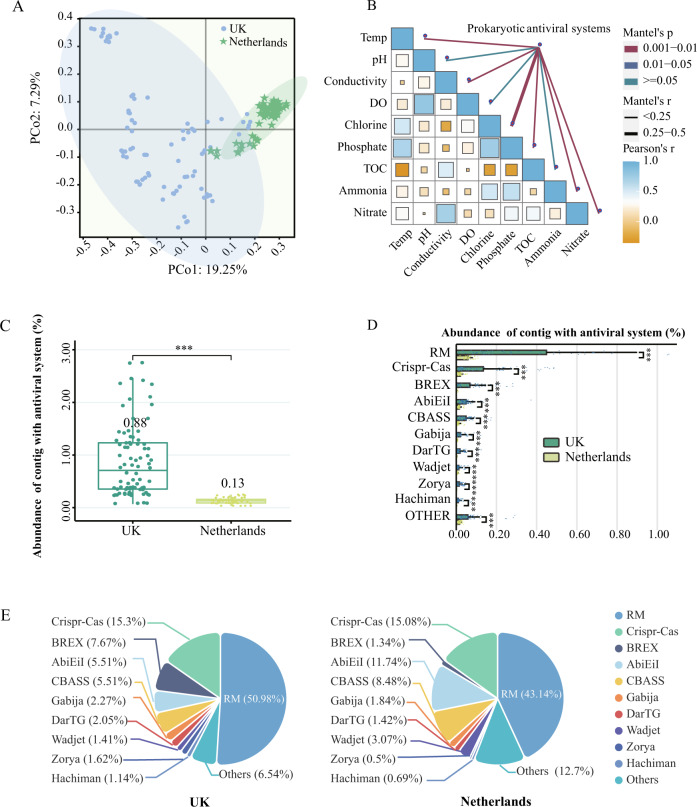
The profile of prokaryotic antiviral systems in DWDS. Drinking water samples were collected from 12 drinking water systems in the UK with disinfectant (*n* = 7, i.e., UK) and the Netherlands without disinfectant (*n* = 5, i.e., Netherlands), and samples were collected at two to four locations in each DWDS. **A** Beta diversity of prokaryotic antiviral systems (PCoA) in DWDS with and without residual disinfectant. PCo1 is 19.25% and PCo2 is 7.29% based on the Bray-Curtis distance. **B** The mental test of prokaryotic antiviral systems and the water quality parameter. **C** The total relative abundance of prokaryotic contigs with antiviral system among all microbial contigs (TPM) in DWDS from the UK (green) and the Netherlands (yellow). **D** The relative abundance of prokaryotic contigs with different antiviral systems among all microbial contigs (TPM) in the UK (green) and Netherlands (yellow) DWDS, and the top ten were listed. **E** The proportion of each defense system in the total defense systems. Asterisks (***) in this study represent significant differences (*P* < 0.001) based on Student’s *T*-test, **P < 0.01 and *P < 0.05.

Prokaryotes have evolved various defense systems to prevent virus infection and prophage activation [8]. DefenseFinder revealed various prokaryotic antiviral systems harbored by the drinking water microbiome [9], and PCoA analysis showed that prokaryotic antiviral systems clustered separately in the presence and absence of disinfectants (Fig. 1A). Moreover, the mental test suggested that chlorine was the predominant driver (rho = 0.29, *p* = 0.001) of the dissimilarity in the composition of prokaryotic antiviral systems (Fig. 1B and Table S4). Specifically, 0.88% of prokaryotic contigs carried antiviral systems in the UK DWDS, which was about seven times higher than that in the Netherlands DWDS (Fig. 1C). The abundance of identified prokaryotic antiviral systems was significantly higher in the UK DWDS microbiome with the restriction-modification (RM) and Clustered Regularly Interspaced Short Palindromic Repeats (CRISPR)-Cas systems enriched to the greatest extent (Fig. 1D and Table S5). The relative abundance of RM and CRISPR-Cas systems was 51% and 15%, respectively, in the UK and 43% and 15%, respectively, in the Netherlands (Fig. 1E). In the UK DWDS microbiome, the bacteriophage exclusion (BREX) (8%) and abortion infection (Abi) systems (6%) were the third and fourth richest antiviral defense systems, respectively. However, in the Netherlands DWDS, Abi system and Cyclic-oligonucleotide-based anti-phage signaling systems (CBASS) were the third (12%) and fourth (9%) richest antiviral defense systems, respectively. These results suggested there was an enhancement of prokaryotic antiviral systems in the drinking water microbiome driven predominately by chlorine.

### Prokaryotic RM and CRISPR-Cas subtype systems in the drinking water microbiomes

**Fig. 2:**
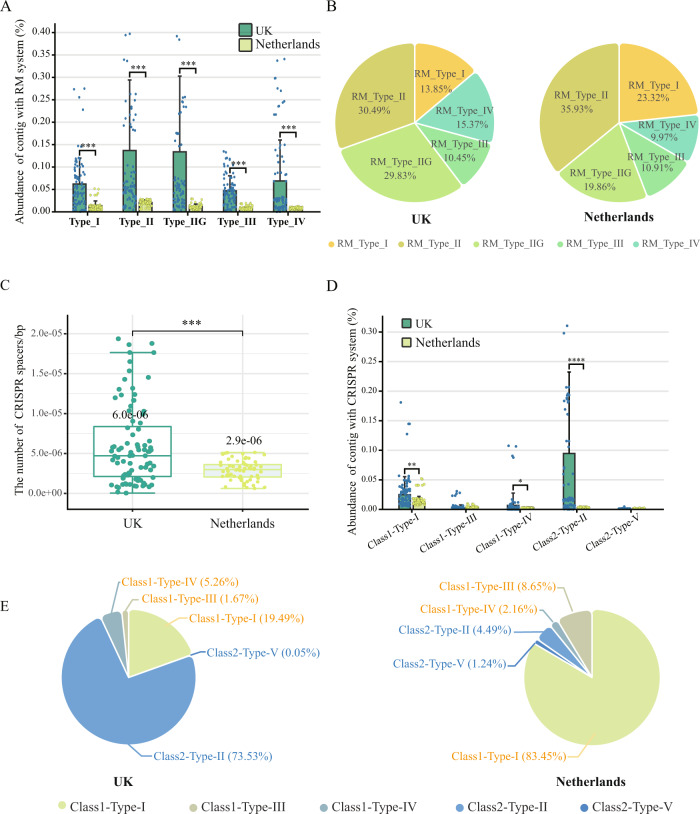
RM and CRISPR-Cas subtype systems of prokaryotes in DWDS. **A** The total relative abundance of prokaryotic contigs with different type RM systems among all microbial contigs (TPM) in the UK (green) and Netherlands (yellow) DWDS. **B** The proportion of RM subtype systems in the total RM system. **C** The number of CISPR spacers per prokaryotic contig carrying CISPR-Cas systems in the UK (green) and Netherlands (yellow) DWDS (standardized based on contig length). **D** The total relative abundance of prokaryotic contigs with different types of CRISPR-Cas systems among all microbial contigs (TPM) in the UK (green) and Netherlands (yellow) DWDS. **E** The proportion of 5 CRISPR-Cas subtype systems in the total CRISPR-Cas system.

The RM system can be divided into four major types (Types I-IV) based on their structural and functional patterns [39]. The relative abundance of RM systems was significantly higher for all the types in the UK microbiome (Fig. 2A). Concretely, Types I and III RM systems consist of complex methyltransferase (MTase) and restriction endonuclease (REase), which require ATP for DNA hydrolysis [39, 40]. The proportion of Type I accounted for 14% and 23% in the UK and Netherlands microbiomes, respectively (Fig. 2B), while there was no significant difference in the proportion of Type III. Type II usually consists of a monomer MTase and a dimeric complex REase, and the simplified structure have a lower metabolic burden [41]. Type II accounted for 60% and 56% of the microbiome in the UK and the Netherlands, respectively. Notably, there was a special Type II system (Type IIG) only contains a single protein with both methyltransferase and endonuclease activities [41]. The proportion of Type IIG was 30% and 20% in the UK and Netherlands microbiomes, respectively. The percentage of Type IIG among all RM systems was significantly positively correlated with the concentration of residual chlorine and phosphate (*p* < 0.01, Fig. S1). Type IV contains only a REase component whose low specificity enables the protection of host cells from a broad range of foreign DNA [42]. The proportion of Type IV was 15% and 10% in the UK and Netherlands microbiomes, respectively. These results suggested that prokaryotic RM defense systems in the UK DWDS exhibited higher community abundance and lower metabolic burden compared to those in the Netherlands DWDS.

CRISPR-Cas immunity, as the major adaptive and heritable defense system in prokaryotic communities, plays an important role in prokaryote-phage coevolution [43]. In the DWDS microbiome, the CRISPR-Cas system was the second most abundant antiviral system (Fig. 2C, D), identifying five types of CRISPR-Cas systems (e.g., types I-V), with types I, II, and IV being significantly more abundant in the UK DWDS compared to the Netherlands (*p* < 0.01, Fig. 2D). Type I can recognize and cleave the phage genome more quickly after it has entered the cell [43, 44], and it was the most prominent with the relative abundance of 83% in the Netherlandish DWDS, while that was only 19% in the UK DWDS. Type II has a more streamlined effector (consisting of protein Cas9 only) and better foreign gene targeting capability, representing low cost and high accuracy of defense [43, 44]. Type II was the dominant system in the UK DWDS with 74%, but that was only 4% in the Netherlands DWDS (Fig. 2E). The percentage of Type II among all CRISPR systems was also significantly positively correlated with the concentration of residual chlorine (*p* < 0.01, Fig. S1). Furthermore, the proportion of Types III, IV, and V was relatively small, at only 2%, 5%, 1% in the UK, and 9%, 2%, 1% in the Netherlands, respectively. The number of CRISPR-Cas spacers (recorded viral sequences) could reflect the defense potential of CRISPR-Cas systems since more spacers could usually result in more extensive phage genome recognition [45]. In the UK DWDS, the average number of spacers contained in each CRISPR-Cas system was twice that of the Netherlands DWDS (Fig. 2C). Consequently, CRISPR system in the Netherlands DWDS microbiome was fast-responding but high metabolic burden, whereas disinfection stress might further select for CRISPR-Cas system with lower fitness cost and broader spectrum in the UK DWDS.

### The carriers of antiviral systems in DWDS prokaryotic community

**Fig. 3:**
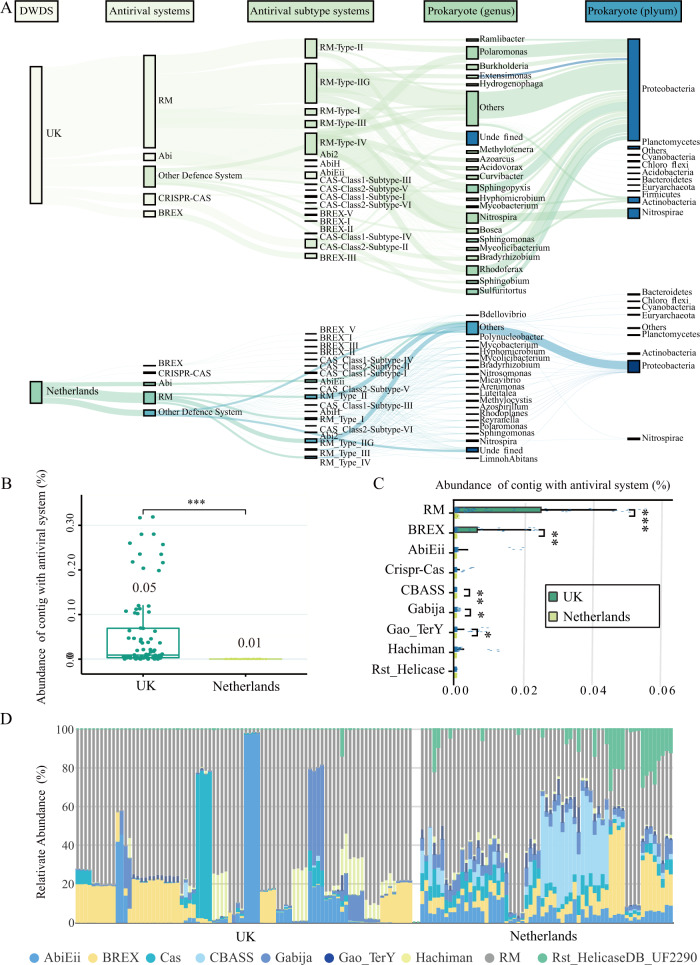
Prokaryotic communities carrying antiviral systems in DWDS. **A** The distribution of antiviral system in prokaryotic community in DWDS from the UK (top) and the Netherlands (bottom). **B** The total relative abundance of lysogenic prokaryotic contigs with antiviral system among all microbial contigs (TPM) in the UK (green) and Netherlands (yellow) DWDS. **C** The relative abundance of lysogenic prokaryotic contigs with different antiviral systems among all microbial contigs (TPM) in the UK (green) and Netherlands (yellow) DWDS. **D** The proportion of each defense system of lysogenic prokaryotic contigs among the total defense system of lysogenic prokaryotic contigs (%).

There were significant differences in the composition of the DWDS microbiome between the UK and the Netherlands (Fig. S2) [15], and so did the major prokaryotes carrying antiviral systems (Fig. 3A and S3). The top 20 prokaryotic genera (59% of all prokaryotes) carried only 29% of the defense systems in the Netherlands DWDS, while which was as high as nearly 73% (distributed in 74% prokaryotes) in the UK DWDS. This means that there was a strong consistency between the main genera carrying antiviral systems and dominant prokaryotes in the UK DWDS, but most antiviral systems were not carried by local dominant prokaryotes in the Netherlands DWDS. Apparently, prokaryotes with antiviral defense systems were more likely to dominate in the more stressful DWDS with nutritional limitations and residual chlorine.

Lysogenized prokaryotes, which carrying prophages, are the main participants in the phage-host symbiosis [13]. The relative abundance of lysogenized prokaryotes in the UK DWDS microbiome was over 100-fold higher than that in the Netherlands DWDS microbiome (Fig. S4). A total of nine antiviral systems were detected in lysogenized prokaryotes of the DWDS microbiome, and their total abundance in the UK was five times higher than that in the Netherlands (Fig. 3B, C). Precisely, RM system was the dominant one, followed by BREX (Fig. 3D), and the percentage of RM and BREX in all systems carried by lysogenized prokaryotes were close to 69% in the Netherlands DWDS, while that was as high as 95% in the UK DWDS (Fig. 3D and S5). These results suggested that some RM and BREX systems might be compatible with prophages.

### Patterns of virome and phage-prokaryote symbiosis in DWDS

**Fig. 4:**
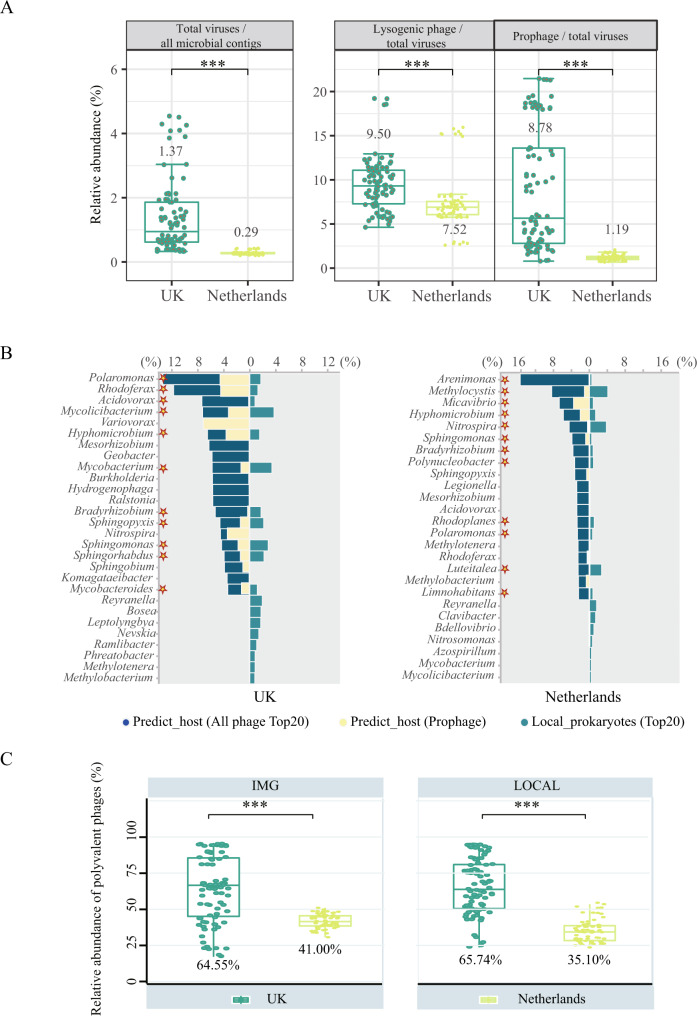
The profile of virome and phage-host interaction dynamics in DWDS. **A** From left to right, the fraction of free viral contigs (identified by “Virsorter2+CheckV+Virsorter2” method) among all microbial contigs; the fraction of lysogenic phages among all viral contigs (identified by Deephage); the fraction of prophage (identified by “CheckV+Virsorter2” method) among all viral contigs. **B** Association between predicted-hosts and local dominant prokaryotic genera in disinfected UK (left) and non-disinfected Netherlands (right) DWDS. The mazarine bars represent the relative abundance of predicted dominant host genera (top 20) in all potential hosts, while the yellow bars represent the ratio of the dominant genera associated with prophages, and the green bars represent the relative abundance of dominant prokaryotic genera (top 20) among all prokaryotes. The asterisks mean that the dominant viral host genus was matched with the dominant prokaryotic genus analyzed by DWDS metagenomic data. **C** The relative abundance of polyvalent phages among all viruses linked to the host in the UK (green) and the Netherlands (yellow) DWDS. Viruses linking with two or more prokaryotic genera were defined as polyvalent phages. The prediction was performed based on IMG/VR database (left) and based on local DWDS metagenomic data (right).

A total of 14,224 free viral contigs were recovered in this study, among which less than 32% were annotated. PCoA analysis revealed that viral communities from different DWDS microbiomes clustered separately (Fig. S6), which was consistent with that of prokaryotic communities [15]. Mantel tests based on viral contigs indicated that chlorine (rho = 0.41, *p* = 0.001) and phosphate (rho = 0.40, *p* = 0.001) were the main drivers of the dissimilarity between the two DWDS viromes (Table S6). In terms of viral diversity (Fig. S7), the Shannon index of DWDS virome in the Netherlands (7.87) was higher than in the UK (5.96, *p* < 0.01). The difference in viral community diversity was consistent with the previous results from Hegarty’s study on the influence of disinfectants on DWDS virome [14]. Moreover, the relative abundance of viral contigs among all microbial contigs in the Netherlands was 0.3%, while that was 1.4% in the UK (Fig. 4A). Additionally, there was a significant positive correlation between the relative abundance of viral contigs and prokaryotic antiviral systems (*r* = 0.97, *p* < 0.01) (Fig. S8), suggesting that the high abundance of prokaryotic antiviral systems in the UK DWDS might be conducive to prokaryotic defense to highly abundant phages.

The fractions of lysogenic phages and lysogenized prokaryotes are hallmarks of prokaryote-phage symbiosis [46]. Viral attribution identified 5801 viral contigs (nearly 40%) as lysogenic phages and prophages, as well as the fraction of lysogenic phages in the UK DWDS virome was substantially higher than that in the Netherlands virome (9.5% vs 7.5%, *p* < 0.01, Fig. 4A). Moreover, the proportion of prophages among all viral contigs in the UK DWDS was 7-fold higher than that in the Netherlands DWDS (*p* < 0.01, Fig. 4A). Consistently, the relative abundance of lysogenized prokaryotes in the UK DWDS microbiome was higher than that in the Netherlands DWDS microbiome (Fig. S5). Pearson correlation analysis showed that chlorine was most significantly positively correlated with phage lysogenicity levels (*r* = 0.36, *p* < 0.01) (Fig. S9). These results show that there was an intense prokaryote-phage symbiosis in the UK DWDS microbiome driven by residual chlorine and nutritional restriction, while this symbiosis was weaker in the Netherlands DWDS microbiome with only oligotrophic stress.

Additionally, Pearson correlation analysis (Fig. S8) showed that Type IIG (*r* = 0.69, 0.76, and 0.64), IV (*r* = 0.36, 0.32, and 0.34) RM systems and Type II CRISPR-Cas system (*r* = 0.83, 0.89 and 0.72, *p* < 0.01) were significantly positively correlated with the abundance of total viruses, prophage and lysogenized prokaryotes (*p* < 0.01), while Type I RM system (*r* = −0.32, −0.4 and −0.36, *p* < 0.01) and Type I CRISPR-Cas system (*r* = −0.68, −0.80 and −0.62, *p* < 0.01) were significantly negatively correlated with them (Fig. S8). This suggested that these prokaryotic antiviral systems had a close association with the prokaryote-phage symbiosis under the environmental stress.

### The intimate linkage of phages to prokaryotes in DWDS microbiome

The prokaryote-phage interactions could also be reflected by the direct linkage between host-virus obtained via “in situ” host prediction [47]. There were 2197 matches established between 642 viral contigs and the taxonomically annotated hosts, with 1837 via tRNA similarity between viral and prokaryotic contigs [32], 71 by matching CRISPR spacers [30, 31] and 289 via identifying lysogenized prokaryotes with integrated prophages, 34 of these viral contigs were simultaneously linked to the host in two different ways (Fig. S10). We focus on the matching between the predicted most probable viral host genera (top 20) and the local predominant genera (top 20). As shown in Fig. 4B, virus-associated host taxa were significantly different between UK and Netherlands DWDS. The proportions of the top 20 matched genera (asterisk) in the UK reached 74% among the predicted viral hosts and 23% of native prokaryotes in microbiome (Fig. 4B), and that in the Netherlands only accounted for 69% among the predicted hosts and 21% among the indigenous prokaryotes (Fig. 4B). Specifically, matched genera by UK virome were mainly represented by dominant bacteria *Polaromonas*, *Sphingopyxis*, *Bradyrhizobium*, and *Rhodoferax*, etc. in the UK DWDS. However, the matched genera in the Netherlands DWDS were mainly represented by dominant bacteria in the Netherlands (e.g., *Methylocystis*, *Nitrospira*, and *Luteitalea*) (Fig. S2). These results suggested that phages in DWDS tend to infect the local dominant prokaryotes. It is noteworthy that the proportion of hosts linked by prophages among the UK DWDS was significantly higher relative to the Netherlands DWDS (28% vs 12%, *P* < 0.01) (Fig. 4B). The intimate linkage of phages to prokaryotes implied that there was a strong reliance of phages on prokaryotes, as well as a significant potential impact of phages on prokaryotes in DWDS.

Phages in harsh environments could infect multiple genera in response to low microbial abundance and activity [48]. Host range prediction based on local metagenomic data and the IMG/VR database [49] concurred that a substantial proportion of viral contigs could be linked to multiple genera with 33% (211/642) based on the local metagenomic data, and 43% (481/1107) based on the IMG/VR database (Fig. S11). In terms of relative abundance, polyvalent phages accounted for 41% (IMG/VR database) and 35% (local metagenomic data) of viral contigs linked to the host in the Netherlands DWDS (Fig. 4C). However, the relative abundance of predicted polyvalent phages both exceed 65% in the UK DWDS, based on IMG/VR database and local metagenomic data. There was a significant positive correlation between the relative abundance of polyvalent phages among phages predicted to host and chlorine concentration (Fig. S9), indicating that polyvalent phages could better cope with the oxidative stress of chlorine [13]. Consequently, the high abundance of polyvalent phages corroborated more intimate prokaryote-phage linkage and synthesis in the UK DWDS. In addition, the abundance of prokaryotic antiviral systems had a significant positive correlation with the proportion of polyvalent phages in all phages linked to host (*r* = 0.80, *p* < 0.01, Fig. S8), suggesting that high antiviral systems might promote the enrichment of polyvalent phages.

### Antiviral systems carried by phage genomes in DWDS

**Fig. 5:**
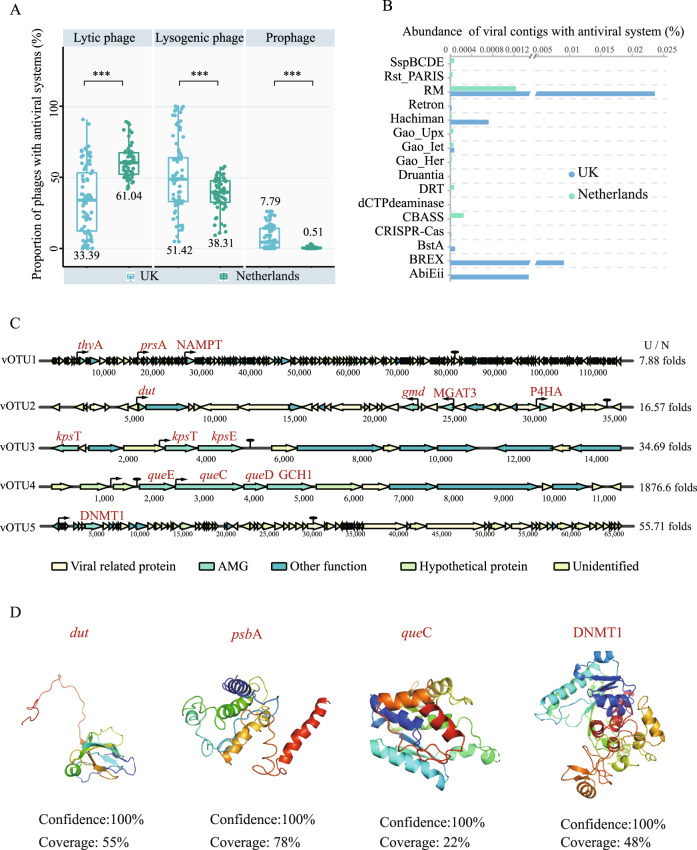
The antiviral systems and AMGs carried by viruses. **A** The percentage of antiviral systems distributed in lytic phages (left), lysogenic phages (middle), and prophages (right) among all viral contigs carrying antiviral systems, respectively. **B** The relative abundance of viral contigs with 16 different antiviral systems among all microbial contigs in DWDS with and without disinfectant. Asterisks represent significant differences (***P < 0.001, **P < 0.01) based on Student’s *T*-test. **C** Genome map of ten viral contigs containing AMGs related to biofilm formation, disinfectant resistance and methylation-modifying enzymes. The thick arrow length within the gene maps corresponds to the size of the open reading frame (ORF). The thin arrows and ovals represent promoters and terminators, respectively. C/N represents the folds of the abundance of the viral contig in disinfected DWDS to non-disinfected DWDS. **D** Structure prediction of AMG-encoded proteins.

Antiviral systems were prokaryotic weapons against phages, interestingly, 16 antiviral systems were detected on phage genomes. Phages carrying antiviral systems were dominated by the RM, BREX, and Abi systems, and the relative abundance in the UK DWDS was 18 times higher than Netherlands DWDS (Fig. S12). Furthermore, almost 60% were distributed on lysogenic phages and prophages in the UK DWDS (Fig. 5A), while only 39% in the Netherlands DWDS. The RM system (especially the Type IIG) was the predominant antiviral system with a relative abundance of over 65%, followed by the BREX system (25%) in the UK DWDS (Fig. 5B). Consequently, the genes encoding the methylation modified protein were also carried by phages, such as DNMT1 encoding DNA (cytosine-5)-methyltransferase (Fig. 5C,D). This may be due to the fact that disinfection stress enriched the prokaryotic community of the UK DWDS with more RM and BREX systems relative to the Netherlands DWDS. These results corroborated that phages are important mediators of the horizontal transfer of antiviral systems among prokaryotic communities [50].

## Discussion

To reveal the association of prokaryotic antiviral systems and symbiotic phage communities, we systematically investigated the profile of prokaryotic antiviral systems, phage communities and prokaryote-phage interactions in microbiomes from the disinfected UK DWDS and the non-disinfected Netherlands DWDS. Chlorine was proved to be not only the main factor shaping prokaryotic and viral communities, but also might cause the variation of prokaryotic antiviral system and the enhancement of prokaryote-phage symbiosis. Antiviral systems are crucial for prokaryotes to mitigate the threat of phage infection [9, 10]. Various antiviral systems were detected in DWDS prokaryotes, and their abundance in prokaryotes was higher in the UK DWDS relative to those in the Netherlands DWDS (Fig. 1C), which satisfied the need for stronger antiviral ability to cope with the more severe infection pressure associated with high viral abundance (Fig. 4A). Moreover, the significant positive correlation between the antiviral system and polyvalent phages advised that the high abundance of antiviral system may also be one of the reasons for the enrichment of polyvalent phages in the UK DWDS (Fig. 4C). In the UK DWDS, prokaryotic antiviral systems were mainly carried by dominant prokaryotes with strong stress resistance (Fig. 2A) [15], which were also the main predicted phage hosts (Fig. 4B). These results suggested that the prokaryotes with antiviral and antioxidant abilities could better survive in the UK DWDS under the combined stress of phage infection and residual chlorine.

RM and CRISPR-Cas systems were the most prevalent antiviral systems in both DWDS (Fig. 1E), while the composition of RM and CRISPR-Cas subtype systems indicated that the antiviral strategies of prokaryotes were different in the UK and Netherlands DWDS microbiome (Fig. 2). Specifically, these systems with higher energy consumption and advanced defense speed were prevalent in the Netherlands DWDS, including Type I and other II RM systems, as well as the Type I CRISPR-Cas system. However, Type IIG and IV RM systems, and the Type II CRISPR-Cas system which exhibited lower fitness cost [51], were enriched in the UK DWDS (Fig. 2). Furthermore, the positive correlation between phages with lysogenic potential and the abundance of Type IIG and IV RM systems, as well as Type II CRISPR-Cas system was observed in DWDS microbiome (Fig. S8), suggesting that these systems might be conducive to prokaryote-phage symbiosis, which reveals the necessity for future attention to the symbiotic mechanisms of different antiviral systems under stress. Additionally, compared with the Netherlands, more CRISPR-Cas spacers were carried by a single prokaryotic cell on average in the UK microbiome, enabling a broader defense spectrum (Fig. 2C). Therefore, compared with the Netherlands, the advantageous prokaryotic antiviral systems in the UK appeared to be more efficient (i.e., low metabolic burden and broad spectrum), which could contribute to the formation and maintenance of prokaryote-phage symbiosis.

Interestingly, several types of antiviral systems were also observed in phage genomes, which were mainly distributed in lysogenic phage genomes and their abundance in the UK DWDS microbiome was higher significantly as compared with the Netherlands DWDS (Fig. 5A and S11). Phage-carried antiviral systems could enhance the protection of host prokaryotes from secondary phage infection, which is important for lysogenic phages to ensure self-resource exclusivity [52]. Moreover, the abundance of systems with methylation potential (e.g., RM and BREX systems) in the UK DWDS was higher than that in the Netherlands DWDS (Fig. 5B), through which phages may avoid being recognized by prokaryotic antiviral systems [50], which is beneficial to promote prokaryote-phage symbiosis in the UK DWDS with disinfectant. In addition, as the mediator of horizontal gene transfer, phages can also help prokaryotes immune to other phages by promoting the enrichment of genes related to the antiviral system [53]. Consequently, RM and BREX systems were the main systems carried by lysogenized prokaryotes (Fig. 3B), and their proportion was enriched with enhanced prokaryote-phage symbiosis, indicating that these systems in lysogenized prokaryotes might be compatible with prophages.

The dynamic interaction between prokaryotic antiviral systems and phages could shape the symbiotic phages. Hegarty et al. reported that the viral AMGs were mainly related to survival and replication of phage in the oligotrophic DWDS without disinfectant. However, genes involved in oxidative stress mitigation were broadly observed in phage genomes in disinfected DWDS [14]. Similar patterns were also identified in our study (Fig. 5C, D). Under the joint influence of chemical stress and antiviral systems, the phages in UK DWDS exhibited greater potential to improve the host environmental adaptability relative to the phages in the Netherlands DWDS. Accordingly, the prokaryotes in the Netherlands DWDS tended to avoid the threat from phage lysis rather than seek the potential benefits provided by viral AMGs. In contrast, prokaryotes in the UK DWDS tended to strengthen prokaryote-phage mutualism through the regulation of the antiviral systems, which resulted in high levels of phage lysogenicity in the UK DWDS microbiome. Taken together, environmental stress and phage infection can affect prokaryotic antiviral systems, which in turn shape the symbiotic phage communities.

## Conclusion

This metagenomic study for the first time investigated the profile of the prokaryotic antiviral systems at the community level and the prokaryote-phage interaction in the DWDS microbiome. The residual chlorine was identified as the main driver for the enhanced antiviral systems and prokaryote-phage symbiosis. The prokaryotic antiviral systems in DWDS with residual disinfectant exhibited higher abundance, efficiency, and broader spectrum relative to those in DWDS without disinfectant. Furthermore, more the antiviral system and AMGs related to oxidative stress mitigation were carried by symbiotic phages in the presence of disinfectant. We corroborated that there was a vital association between prokaryotic antiviral systems and their symbiotic phages, and this association could be crucial for prokaryotic survival in DWDS. Our study provides new insights into microbial adaptation in hostile conditions and may inspire ecologically-informed strategies for microbiome manipulation in DWDS.

## Supplementary Information

Supporting Information

## Data Availability

All metagenomic sequence data of DWDS microbiome in this study were collected from NCBI and affiliated with bioproject number PRJNA533545.

## References

[1] Sokol NW, Slessarev E, Marschmann GL, Nicolas A, Blazewicz SJ, Brodie EL (2022). Life and death in the soil microbiome: how ecological processes influence biogeochemistry. Nat Rev Microbiol.

[2] Kuypers MMM, Marchant HK, Kartal B (2018). The microbial nitrogen-cycling network. Nat Rev Microbiol.

[3] Prosser JI (2015). Dispersing misconceptions and identifying opportunities for the use of ‘omics’ in soil microbial ecology. Nat Rev Microbiol.

[4] Kazamia E, Helliwell KE, Purton S, Smith AG (2016). How mutualisms arise in phytoplankton communities: building eco-evolutionary principles for aquatic microbes. Ecol Lett.

[5] Bikel S, Valdez-Lara A, Cornejo-Granados F, Rico K, Canizales-Quinteros S, Soberon X (2015). Combining metagenomics, metatranscriptomics and viromics to explore novel microbial interactions: towards a systems-level understanding of human microbiome. Comput Struct Biotec.

[6] Roossinck MJ (2011). The good viruses: viral mutualistic symbioses. Nat Rev Microbiol.

[7] Warwick-Dugdale J, Buchholz HH, Allen MJ, Temperton B (2019). Host-hijacking and planktonic piracy: how phages command the microbial high seas. Virol J.

[8] Stern A, Sorek R (2011). The phage-host arms race: shaping the evolution of microbes. Bioessays.

[9] Tesson F, Herve A, Mordret E, Touchon M, d’Humieres C, Cury J (2022). Systematic and quantitative view of the antiviral arsenal of prokaryotes. Nat Commun.

[10] Doron S, Melamed S, Ofir G, Leavitt A, Lopatina A, Keren M (2018). Systematic discovery of antiphage defense systems in the microbial pangenome. Science.

[11] Tesson F, Bernheim A (2023). Synergy and regulation of antiphage systems: toward the existence of a bacterial immune system?. Curr Opin Microbiol.

[12] Zheng XX, Jahn MT, Sun MM, Friman VP, Balcazar JL, Wang JF (2022). Organochlorine contamination enriches virus-encoded metabolism and pesticide degradation associated auxiliary genes in soil microbiomes. ISME J.

[13] Huang D, Yu P, Ye M, Schwarz C, Jiang X, Alvarez PJJ (2021). Enhanced mutualistic symbiosis between soil phages and bacteria with elevated chromium-induced environmental stress. Microbiome.

[14] Hegarty B, Dai ZH, Raskin L, Pinto A, Wigginton K, Duhaime M (2022). A snapshot of the global drinking water virome: Diversity and metabolic potential vary with residual disinfectant use. Water Res.

[15] Dai Z, Sevillano-Rivera MC, Calus ST, Bautista-de los Santos QM, Eren AM, van der Wielen P (2020). Disinfection exhibits systematic impacts on the drinking water microbiome. Microbiome.

[16] Pinto AJ, Xi CW, Raskin L (2012). Bacterial community structure in the drinking water microbiome is governed by filtration processes. Environ Sci Technol.

[17] Volk C, Dundore E, Schiermann J, Lechevallier M (2000). Practical evaluation of iron corrosion control in a drinking water distribution system. Water Res.

[18] Fiancette R, Finlay CM, Willis C, Bevington SL, Soley J, Ng STH (2021). Reciprocal transcription factor networks govern tissue-resident ILC3 subset function and identity. Nat Immunol.

[19] Sewe SO, Silva G, Sicat P, Seal SE, Visendi P (2022). Trimming and validation of illumina short reads using trimmomatic, trinity assembly, and assessment of RNA-Seq Data. Methods Mol Biol.

[20] Li DH, Luo RB, Liu CM, Leung CM, Ting HF, Sadakane K (2016). MEGAHIT v1.0: a fast and scalable metagenome assembler driven by advanced methodologies and community practices. Methods.

[21] Gurevich A, Saveliev V, Vyahhi N, Tesler G (2013). QUAST: quality assessment tool for genome assemblies. Bioinformatics.

[22] Guo JR, Bolduc B, Zayed AA, Varsani A, Dominguez-Huerta G, Delmont TO, et al. VirSorter2: a multi-classifier, expert-guided approach to detect diverse DNA and RNA viruses. Microbiome. 2021;9:37. 10.1186/s40168-020-00990-y PMC7852108 33522966

[23] Nayfach S, Camargo AP, Schulz F, Eloe-Fadrosh E, Roux S, Kyrpides NC. CheckV assesses the quality and completeness of metagenome-assembled viral genomes. Nat Biotechnol. 2021;39:578–85. 10.1038/s41587-020-00774-7 PMC8116208 33349699

[24] Fu LM, Niu BF, Zhu ZW, Wu ST, Li WZ. CD-HIT: accelerated for clustering the next-generation sequencing data. Bioinformatics. 2012;28:3150–2. 10.1093/bioinformatics/bts565 PMC3516142 23060610

[25] Wu SF, Fang ZC, Tan J, Li M, Wang CH, Guo Q, et al. DeePhage: distinguishing virulent and temperate phage-derived sequences in metavirome data with a deep learning approach. Gigascience. 2021;10:giab056. 10.1093/gigascience/giab056 PMC8427542 34498685

[26] Hyatt D, Chen G-L, LoCascio PF, Land ML, Larimer FW, Hauser LJ (2010). Prodigal: prokaryotic gene recognition and translation initiation site identification. BMC Bioinformatics.

[27] Camarillo-Guerrero LF, Almeida A, Rangel-Pineros G, Finn RD, Lawley TD (2021). Massive expansion of human gut bacteriophage diversity. Cell..

[28] Sayers EW, Cavanaugh M, Clark K, Pruitt KMD, Schoch CL, Sherry ST (2022). GenBank. Nucleic Acids Res.

[29] Buchfink B, Xie C, Huson DH (2015). Fast and sensitive protein alignment using DIAMOND. Nat Methods.

[30] Russel J, Pinilla-Redondo R, Mayo-Munoz D, Shah SA, Sorensen SJ (2020). CRISPRCastyper: automated identification, annotation, and classification of CRISPR-Cas loci. Crispr J.

[31] Zhang RS, Mirdita M, Karin EL, Norroy C, Galiez C, Soding J (2021). SpacePHARER: sensitive identification of phages from CRISPR spacers in prokaryotic hosts. Bioinformatics.

[32] Chan PP, Lin BY, Mak AJ, Lowe TM (2021). tRNAscan-SE 2.0: improved detection and functional classification of transfer RNA genes. Nucleic Acids Res.

[33] Couvin D, Bernheim A, Toffano-Nioche C, Touchon M, Michalik J, Neron B (2018). CRISPRCasFinder, an update of CRISRFinder, includes a portable version, enhanced performance and integrates search for Cas proteins. Nucleic Acids Res.

[34] Shaffer M, Borton MA, McGivern BB, Zayed AA, La Rosa SL, Solden LM (2020). DRAM for distilling microbial metabolism to automate the curation of microbiome function. Nucleic Acids Res.

[35] Kieft K, Zhou ZC, Anantharaman K (2020). VIBRANT: automated recovery, annotation and curation of microbial viruses, and evaluation of viral community function from genomic sequences. Microbiome.

[36] Li H, Durbin R (2009). Fast and accurate short read alignment with Burrows-Wheeler transform. Bioinformatics.

[37] Villanueva RAM, Chen ZJ. ggplot2: Elegant Graphics for Data Analysis, 2nd edition. Measurement-Interdisciplinary Research and Perspectives. 2019;17:160–7.

[38] Dixon P (2003). VEGAN, a package of R functions for community ecology. J Veg Sci.

[39] Ershova AS, Rusinov IS, Spirin SA, Karyagina AS, Alexeevski AV (2015). Role of restriction-modification systems in prokaryotic evolution and ecology. Biochemistry (Mosc).

[40] Butterer A, Pernstich C, Smith RM, Sobott F, Szczelkun MD, Toth J (2014). Type III restriction endonucleases are heterotrimeric: comprising one helicase-nuclease subunit and a dimeric methyltransferase that binds only one specific DNA. Nucleic Acids Res.

[41] Pingoud A, Wilson GG, Wende W (2014). Type II restriction endonucleases-a historical perspective and more. Nucleic Acids Res.

[42] Loenen WAM, Raleigh EA (2014). The other face of restriction: modification-dependent enzymes. Nucleic Acids Res.

[43] Watson BNJ, Steens JA, Staals RHJ, Westra ER, van Houte S (2021). Coevolution between bacterial CRISPR-Cas systems and their bacteriophages. Cell Host Microbe.

[44] Nussenzweig PM, McGinn J, Marraffini LA (2019). Cas9 cleavage of viral genomes primes the acquisition of new immunological memories. Cell Host Microbe.

[45] Nicholson TJ, Jackson SA, Croft BI, Staals RHJ, Fineran PC, Brown CM (2019). Bioinformatic evidence of widespread priming in type I and II CRISPR-Cas systems. Rna Biol.

[46] Feiner R, Argov T, Rabinovich L, Sigal N, Borovok I, Herskovits AA (2015). A new perspective on lysogeny: prophages as active regulatory switches of bacteria. Nat Rev Microbiol.

[47] Tang TQ, Hou SW, Fuhrman JA, Sun FZ (2022). Phage-bacterial contig association prediction with a convolutional neural network. Bioinformatics..

[48] Goodridge L, Fong K, Wang SY, Delaquis P (2018). Bacteriophage-based weapons for the war against foodborne pathogens. Curr Opin Food Sci.

[49] Roux S, Paez-Espino D, Chen IMA, Palaniappan K, Ratner A, Chu K (2021). IMG/VR v3: an integrated ecological and evolutionary framework for interrogating genomes of uncultivated viruses. Nucleic Acids Res.

[50] Weigele P, Raleigh EA (2016). Biosynthesis and function of modified bases in bacteria and their viruses. Chem Rev.

[51] Azam AH, Tanji Y (2019). Bacteriophage-host arm race: an update on the mechanism of phage resistance in bacteria and revenge of the phage with the perspective for phage therapy. Appl Microbiol Biotechnol.

[52] Biggs KRH, Bailes CL, Scott L, Wichman HA, Schwartz EJ (2021). Ecological approach to understanding superinfection inhibition in bacteriophage. Viruses-Basel.

[53] Rousset F, Depardieu F, Miele S, Dowding J, Laval AL, Lieberman E (2022). Phages and their satellites encode hotspots of antiviral systems. Cell Host Microbe.

