# Supplementary material for: The association of prokaryotic antiviral systems and symbiotic phage communities in drinking water microbiomes
Source: ISME Commun. 2023 May 4;3:46. doi: 10.1038/s43705-023-00249-1 (PMC10160068; doi:10.1038/s43705-023-00249-1)
Supplement: Supplementary file 1 — Supporting Information [file 43705_2023_249_MOESM1_ESM.docx]

**Supporting Information**

**The Association of Prokaryotic Antiviral Systems and Symbiotic Phage Communities in Drinking Water Microbiomes**

Dan Huang^1^, Mengting Maggie Yuan^2^, Juhong Chen^3^, Xiaoxuan Zheng^1^,

Dongsheng Wong^1^, Pedro J.J. Alvarez^4^, Pingfeng Yu^1, *^

^1^ College of Environmental and Resource Sciences, Zhejiang University, Hangzhou, China

^2^ Department of Environmental Science, Policy, and Management, University of California, Berkeley, CA, USA

^3^ Department of Biological Systems Engineering, Virginia Tech, Blacksburg, USA

^4^ Department of Civil and Environmental Engineering, Rice University, Houston, USA

^*^ Corresponding Author:

Email: [yupf@zju.edu](mailto:yupf@zju.edu).cn

ORCID: [0000-0003-0402-773X](http://orcid.org/0000-0003-0402-773X)

**This file runs 21 pages including:**

Figures S1 to S12;

Tables S1 to S6.

This supporting information includes:

**Figures**

Fig. S1 Pearson correlation analysis of relative abundance of RM and CRISPR subtype systems and water quality parameters in DWDS

Fig. S2 The prokaryotic profile in genus level in different DWDS

Fig. S3 The dominant prokaryotic genera (Top 20) carrying with the antiviral system in DWDS

Fig. S4 The fraction of lysogenized prokaryotes among all prokaryotic contigs

Fig. S5 The proportion of each defense system in the total defense system carried by lysogenized prokaryotes (%)

Fig. S6 Beta diversity of viral communities (PCoA) in DWDS with and without residual disinfectant

Fig. S7 Alpha diversity of viral communities in DWDS with and without disinfectant

Fig. S8 Pearson correlation analysis of prokaryotic antiviral systems and phage lysogenicity level

Fig. S9 Pearson correlation analysis of the water quality parameter and phage lysogenicity level

Fig. S10 Phage-host linkages

Fig. S11 The proportion of broad and narrow host range phages predicted based on IMG/VR database (bottom) and local DWDS metagenomic data (top)

Fig. S12 The total relative abundance of viral contigs with antiviral systems among all microbial contigs in DWDS with and without disinfectant

**Tables**

Table S1. The information of the samples collected as part of this study

Table S2. Assembly quality of contigs assessed with QUAST

Table. S3. Quality of viral contigs assessed with QUAST

Table S4. The correlation analysis of the water quality parameter and prokaryotic antiviral systems by Mantel test

Table S5. 46 types of antiviral systems were identified

Table S6. The correlation analysis of the water quality parameter and virome by Mantel test


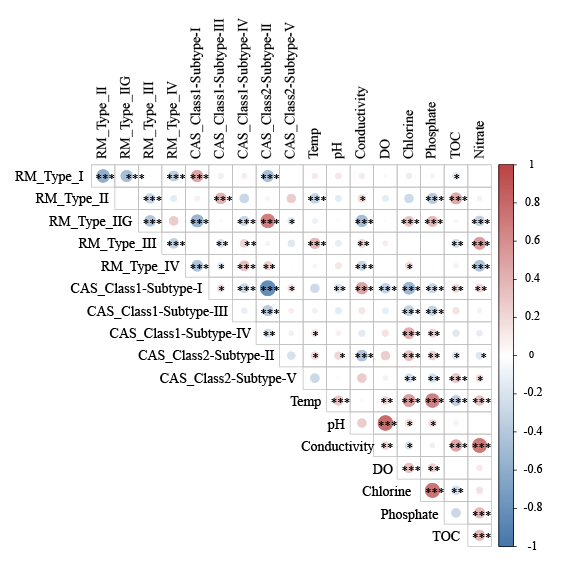


**Figure S1.** Pearson correlation analysis of relative abundance of RM and CRISPR subtype systems and water quality parameters in DWDS


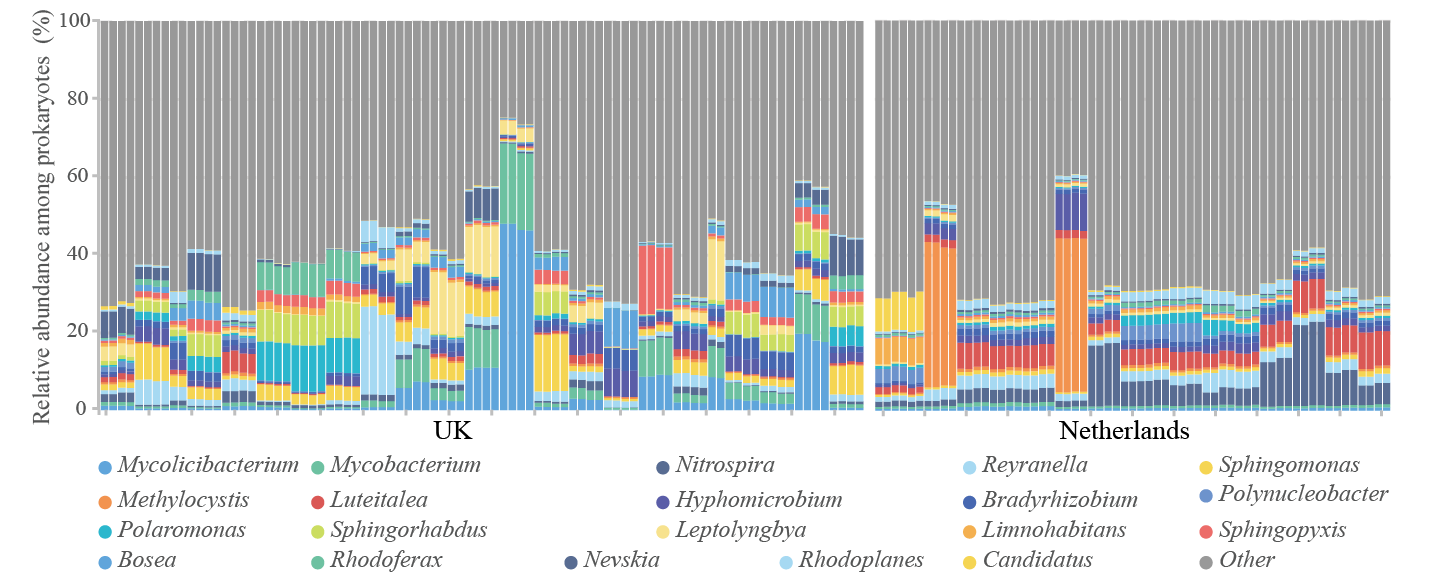


**Figure S2.** The prokaryotic profile in genus level in different DWDS.


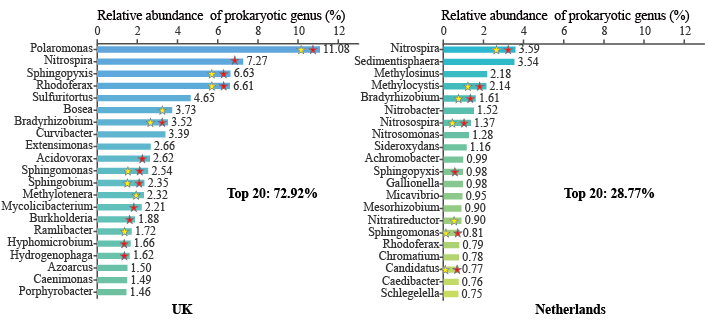


**Figure S3.** The dominant prokaryotic genera (Top 20) carrying with the antiviral system in DWDS. The yellow asterisks mean that these genera were matched with the dominant prokaryotic genus (TOP 20%) analyzed by DWDS metagenomic data, and the red asterisks mean that these genus were matched with the dominant viral host (TOP 20%).


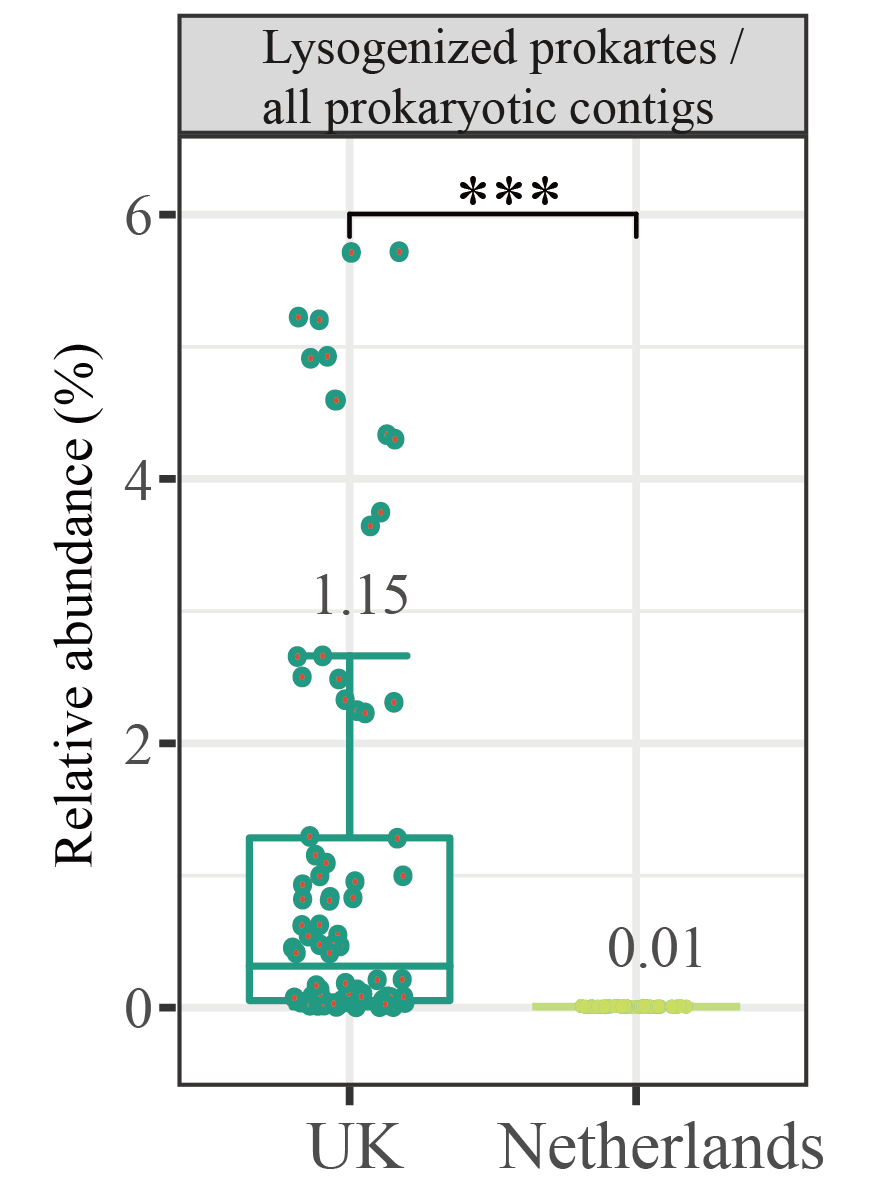


**Figure S4**. The fraction of lysogenized prokaryotes among all prokaryotic contigs


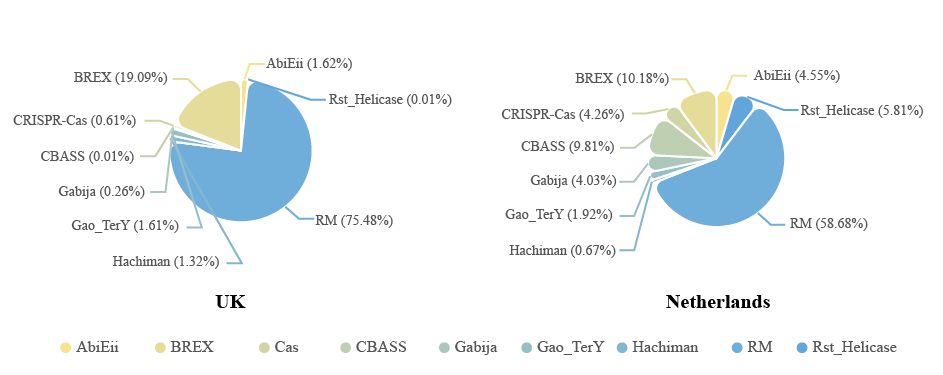


**Figure S5.** The proportion of each defense system (%) in the total defense system carried by lysogenized prokaryotes.


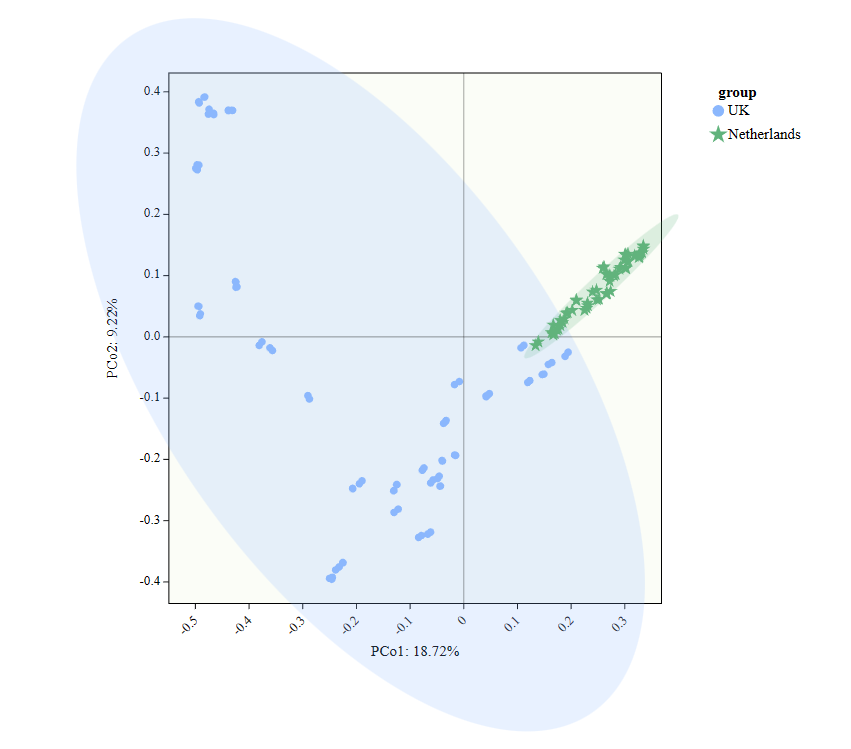


**Figure S6.** Beta diversity of viral communities (PCoA) in DWDS with and without residual disinfectant. PCo1 is 18.72% and PCo2 is 9.22% based on the Bray-Curtis distance.


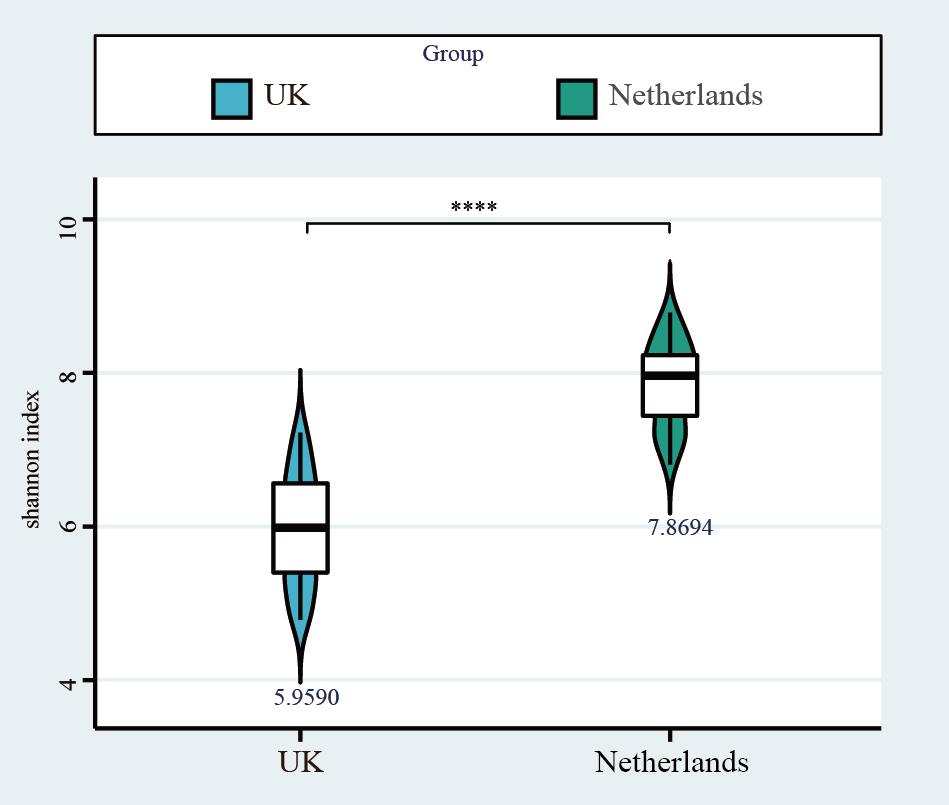


**Figure S7.** Alpha diversity of viral communities in DWDS with and without disinfectant. Asterisks (***) represent the significant difference (*P* < 0.0001) based on Student’s T-test.


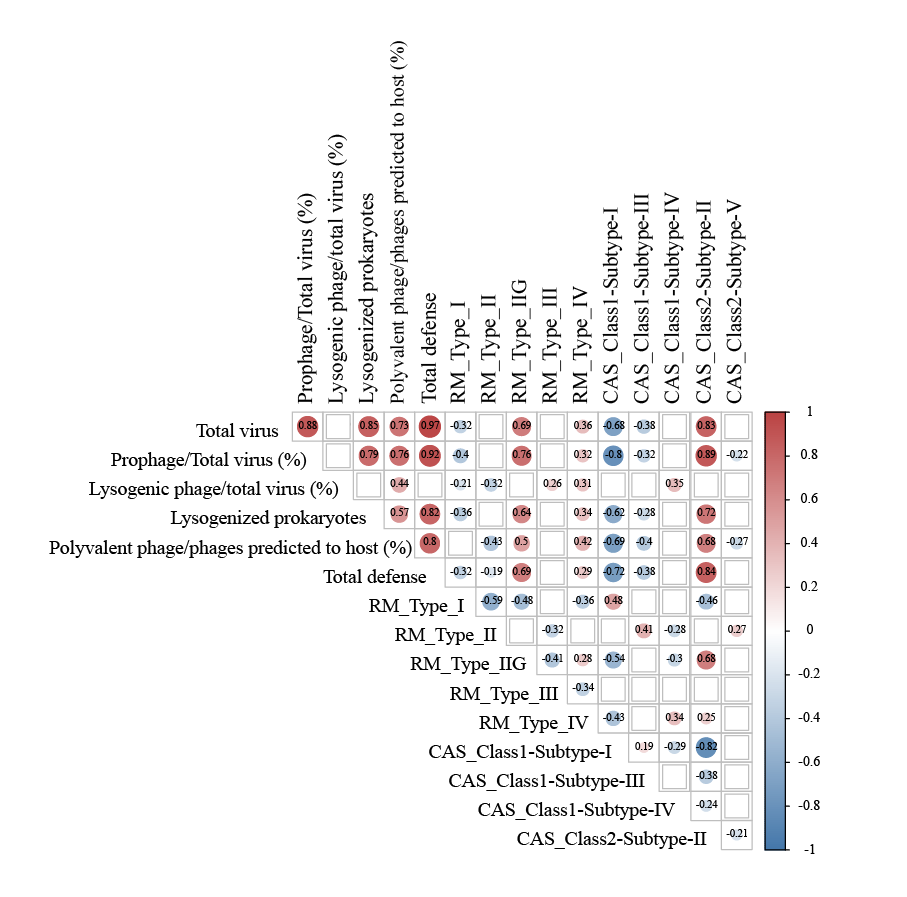


**Figure S8.** Pearson correlation analysis of prokaryotic antiviral systems and phage lysogenicity level.


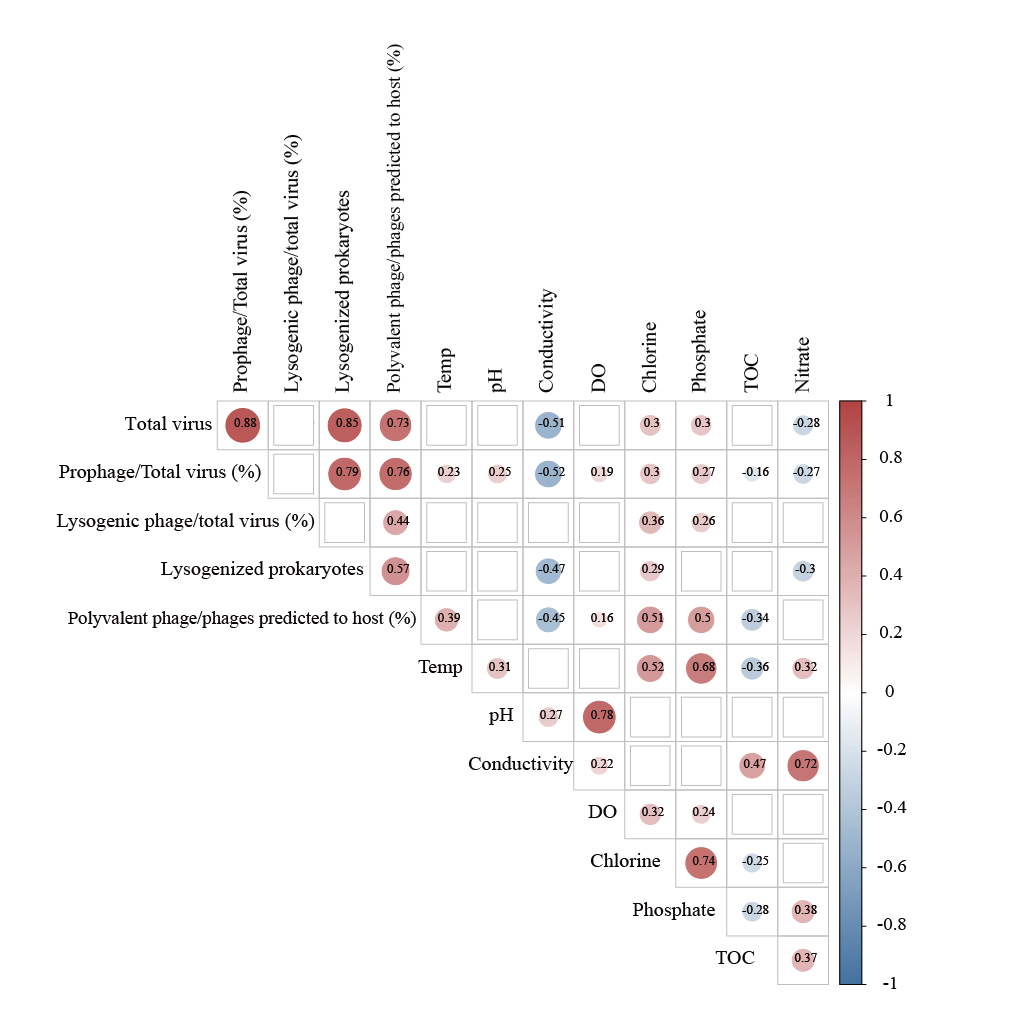


**Figure S9.** Pearson correlation analysis of the water quality parameter and phage lysogenicity level.


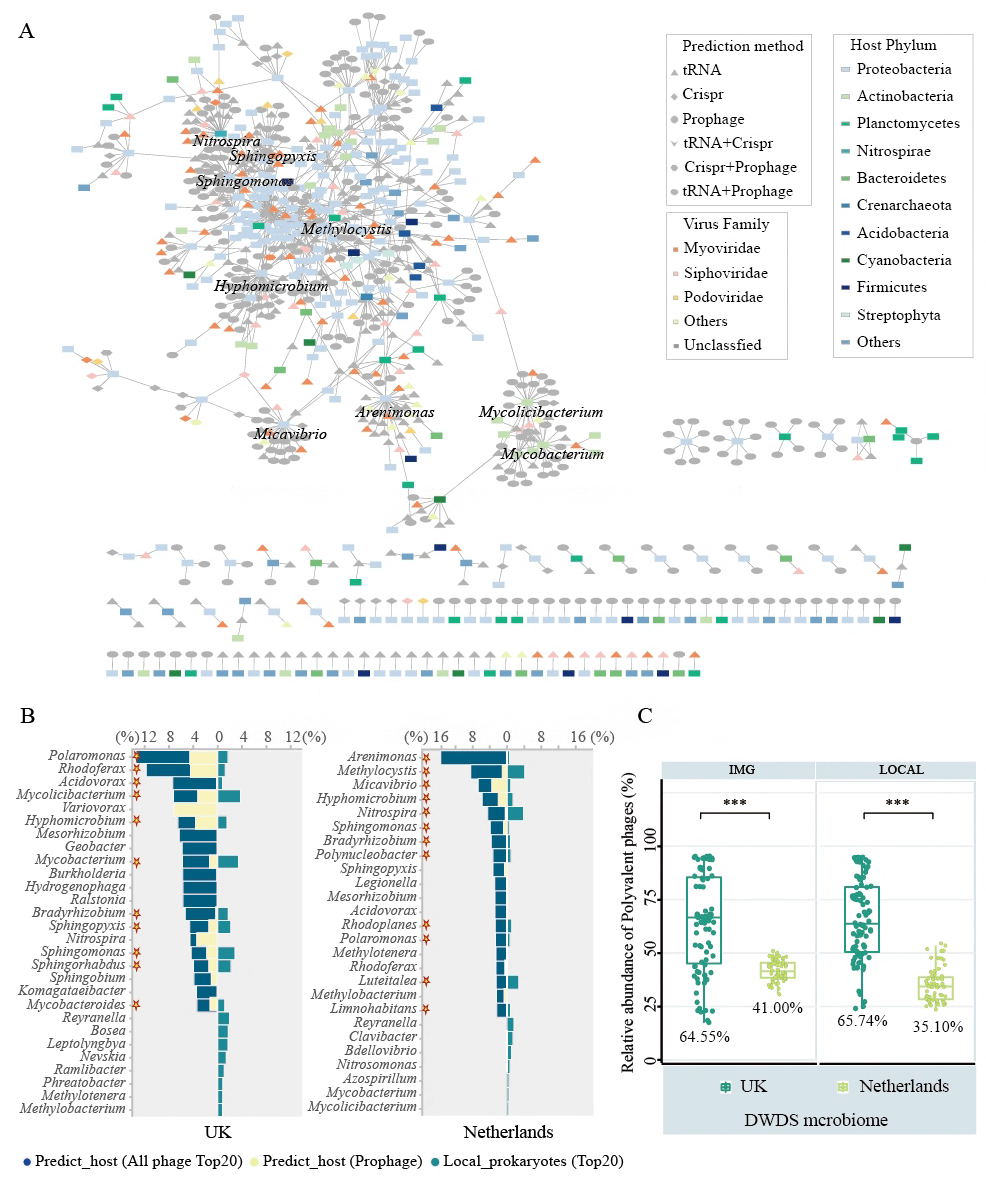


**Figure S10.** Phage-host interaction dynamics. (**A**) Predicted phage–host linkages based on local metagenomic data via tRNA similarity alignment, CRISPR spacer similarity alignment and the prophage linked prokaryotes. The rectangles represent potential host prokaryotes with each rectangle representing one genus, and the colors of the rectangles represent the phylum classification of the host prokaryotes. The dots represent viral contigs, and their colors mean viral annotation at family level.


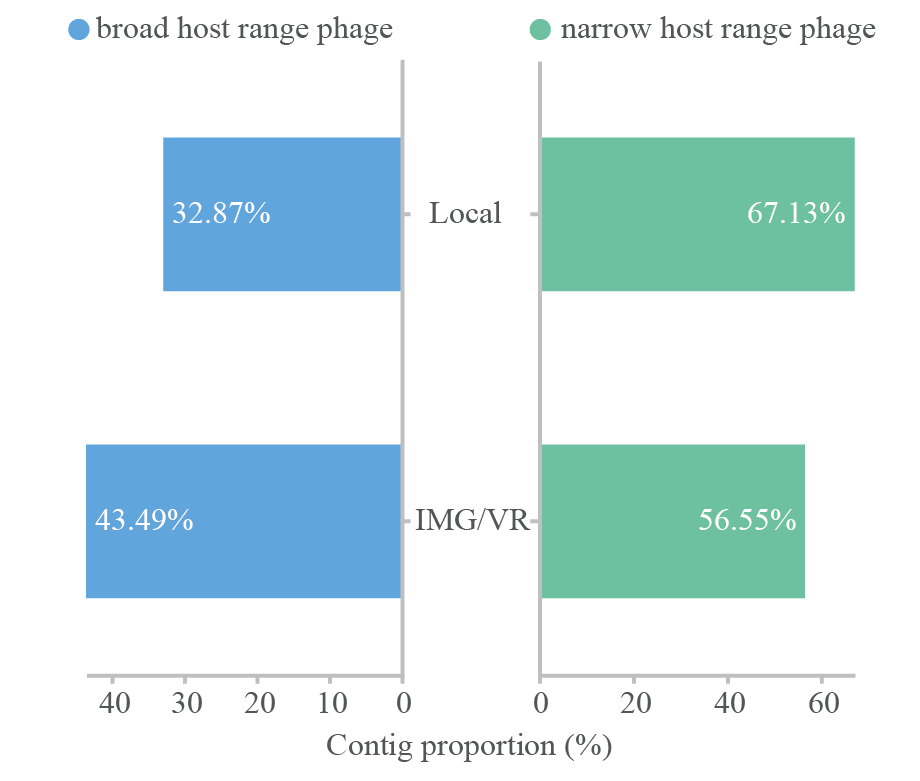


**Figure S11.** The proportion of broad and narrow host range phages predicted based on IMG/VR database (bottom) and local DWDS metagenomic data (top).


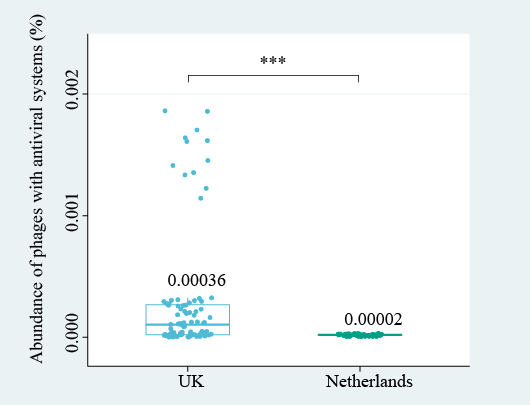


**Figure S12.** The total relative abundance of viral contigs with antiviral systems among all microbial contigs in DWDS with and without disinfectant.

**Table S1**. Samples collected for this study (cited from NCBI bioproject number PRJNA533545).

| sample | DWDS | Type | Country | City | sample | DWDS | Type | Country | City |
| --- | --- | --- | --- | --- | --- | --- | --- | --- | --- |
| UK1-2-1 | UK1 | Chlorinated | UK | London | N1-1-1 | N1 | Not-disinfected | Netherlands | Amsterdam |
| UK1-2-2 | UK1 | Chlorinated | UK | London | N1-1-2 | N1 | Not-disinfected | Netherlands | Amsterdam |
| UK1-2-3 | UK1 | Chlorinated | UK | London | N1-1-3 | N1 | Not-disinfected | Netherlands | Amsterdam |
| UK1-2-4 | UK1 | Chlorinated | UK | London | N1-1-4 | N1 | Not-disinfected | Netherlands | Amsterdam |
| UK1-3-1 | UK1 | Chlorinated | UK | London | N1-2-1 | N1 | Not-disinfected | Netherlands | Amsterdam |
| UK1-3-2 | UK1 | Chlorinated | UK | London | N1-2-4 | N1 | Not-disinfected | Netherlands | Amsterdam |
| UK1-4-1 | UK1 | Chlorinated | UK | London | N1-3-1 | N1 | Not-disinfected | Netherlands | Amsterdam |
| UK1-4-2 | UK1 | Chlorinated | UK | London | N1-3-2 | N1 | Not-disinfected | Netherlands | Amsterdam |
| UK1-4-3 | UK1 | Chlorinated | UK | London | N1-3-3 | N1 | Not-disinfected | Netherlands | Amsterdam |
| UK1-4-4 | UK1 | Chlorinated | UK | London | N1-3-4 | N1 | Not-disinfected | Netherlands | Amsterdam |
| UK2-1-1 | UK2 | Chlorinated | UK | London | N1-4-1 | N1 | Not-disinfected | Netherlands | Amsterdam |
| UK2-1-2 | UK2 | Chlorinated | UK | London | N1-4-2 | N1 | Not-disinfected | Netherlands | Amsterdam |
| UK2-1-3 | UK2 | Chlorinated | UK | London | N1-4-3 | N1 | Not-disinfected | Netherlands | Amsterdam |
| UK2-1-4 | UK2 | Chlorinated | UK | London | N1-4-4 | N1 | Not-disinfected | Netherlands | Amsterdam |
| UK2-2-1 | UK2 | Chlorinated | UK | London | N2-1-1 | N2 | Not-disinfected | Netherlands | Amsterdam |
| UK2-2-2 | UK2 | Chlorinated | UK | London | N2-1-2 | N2 | Not-disinfected | Netherlands | Amsterdam |
| UK2-2-3 | UK2 | Chlorinated | UK | London | N2-1-3 | N2 | Not-disinfected | Netherlands | Amsterdam |
| UK2-2-4 | UK2 | Chlorinated | UK | London | N2-1-4 | N2 | Not-disinfected | Netherlands | Amsterdam |
| UK2-3-1 | UK2 | Chlorinated | UK | London | N2-2-1 | N2 | Not-disinfected | Netherlands | Amsterdam |
| UK2-3-2 | UK2 | Chlorinated | UK | London | N2-2-2 | N2 | Not-disinfected | Netherlands | Amsterdam |
| UK2-3-3 | UK2 | Chlorinated | UK | London | N2-2-3 | N2 | Not-disinfected | Netherlands | Amsterdam |
| UK2-3-4 | UK2 | Chlorinated | UK | London | N2-2-4 | N2 | Not-disinfected | Netherlands | Amsterdam |
| UK2-4-1 | UK2 | Chlorinated | UK | London | N2-3-1 | N2 | Not-disinfected | Netherlands | Amsterdam |
| UK2-4-2 | UK2 | Chlorinated | UK | London | N2-3-2 | N2 | Not-disinfected | Netherlands | Amsterdam |
| UK2-4-3 | UK2 | Chlorinated | UK | London | N2-3-3 | N2 | Not-disinfected | Netherlands | Amsterdam |
| UK2-4-4 | UK2 | Chlorinated | UK | London | N2-3-4 | N2 | Not-disinfected | Netherlands | Amsterdam |
| UK3-1-1 | UK3 | Chlorinated | UK | London | N3-1-1 | N3 | Not-disinfected | Netherlands | Amsterdam |
| UK3-1-2 | UK3 | Chlorinated | UK | London | N3-1-2 | N3 | Not-disinfected | Netherlands | Amsterdam |
| UK3-1-3 | UK3 | Chlorinated | UK | London | N3-1-3 | N3 | Not-disinfected | Netherlands | Amsterdam |
| UK3-1-4 | UK3 | Chlorinated | UK | London | N3-1-4 | N3 | Not-disinfected | Netherlands | Amsterdam |
| UK3-2-1 | UK3 | Chlorinated | UK | London | N3-2-1 | N3 | Not-disinfected | Netherlands | Amsterdam |
| UK3-2-2 | UK3 | Chlorinated | UK | London | N3-2-2 | N3 | Not-disinfected | Netherlands | Amsterdam |
| UK3-2-3 | UK3 | Chlorinated | UK | London | N3-3-1 | N3 | Not-disinfected | Netherlands | Amsterdam |
| UK3-2-4 | UK3 | Chlorinated | UK | London | N3-3-2 | N3 | Not-disinfected | Netherlands | Amsterdam |
| UK4-1-1 | UK4 | Chlorinated | UK | London | N3-3-3 | N3 | Not-disinfected | Netherlands | Amsterdam |
| UK4-1-2 | UK4 | Chlorinated | UK | London | N3-3-4 | N3 | Not-disinfected | Netherlands | Amsterdam |
| UK4-1-3 | UK4 | Chlorinated | UK | London | N3-4-1 | N3 | Not-disinfected | Netherlands | Amsterdam |
| UK4-1-4 | UK4 | Chlorinated | UK | London | N3-4-2 | N3 | Not-disinfected | Netherlands | Amsterdam |
| UK4-2-1 | UK4 | Chlorinated | UK | London | N3-4-3 | N3 | Not-disinfected | Netherlands | Amsterdam |
| UK4-2-2 | UK4 | Chlorinated | UK | London | N3-4-4 | N3 | Not-disinfected | Netherlands | Amsterdam |
| UK4-2-3 | UK4 | Chlorinated | UK | London | N4-1-3 | N4 | Not-disinfected | Netherlands | Amsterdam |
| UK4-2-4 | UK4 | Chlorinated | UK | London | N4-1-4 | N4 | Not-disinfected | Netherlands | Amsterdam |
| UK4-3-1 | UK4 | Chlorinated | UK | London | N4-2-3 | N4 | Not-disinfected | Netherlands | Amsterdam |
| UK4-3-2 | UK4 | Chlorinated | UK | London | N4-2-4 | N4 | Not-disinfected | Netherlands | Amsterdam |
| UK4-3-3 | UK4 | Chlorinated | UK | London | N4-3-1 | N4 | Not-disinfected | Netherlands | Amsterdam |
| UK4-3-4 | UK4 | Chlorinated | UK | London | N4-3-2 | N4 | Not-disinfected | Netherlands | Amsterdam |
| UK4-4-1 | UK4 | Chlorinated | UK | London | N4-3-3 | N4 | Not-disinfected | Netherlands | Amsterdam |
| UK4-4-2 | UK4 | Chlorinated | UK | London | N5-1-1 | N5 | Not-disinfected | Netherlands | Amsterdam |
| UK4-4-3 | UK4 | Chlorinated | UK | London | N5-1-2 | N5 | Not-disinfected | Netherlands | Amsterdam |
| UK4-4-4 | UK4 | Chlorinated | UK | London | N5-1-3 | N5 | Not-disinfected | Netherlands | Amsterdam |
| UK5-1-1 | UK5 | Chlorinated | UK | London | N5-1-4 | N5 | Not-disinfected | Netherlands | Amsterdam |
| UK5-1-2 | UK5 | Chlorinated | UK | London | N5-2-1 | N5 | Not-disinfected | Netherlands | Amsterdam |
| UK5-1-3 | UK5 | Chlorinated | UK | London | N5-2-2 | N5 | Not-disinfected | Netherlands | Amsterdam |
| UK5-1-4 | UK5 | Chlorinated | UK | London | N5-2-3 | N5 | Not-disinfected | Netherlands | Amsterdam |
| UK5-2-1 | UK5 | Chlorinated | UK | London | N5-2-4 | N5 | Not-disinfected | Netherlands | Amsterdam |
| UK5-2-2 | UK5 | Chlorinated | UK | London | N5-3-1 | N5 | Not-disinfected | Netherlands | Amsterdam |
| UK5-2-3 | UK5 | Chlorinated | UK | London | N5-3-2 | N5 | Not-disinfected | Netherlands | Amsterdam |
| UK5-2-4 | UK5 | Chlorinated | UK | London | N5-3-3 | N5 | Not-disinfected | Netherlands | Amsterdam |
| UK5-3-1 | UK5 | Chlorinated | UK | London | N5-3-4 | N5 | Not-disinfected | Netherlands | Amsterdam |
| UK5-3-2 | UK5 | Chlorinated | UK | London | N5-4-1 | N5 | Not-disinfected | Netherlands | Amsterdam |
| UK5-3-3 | UK5 | Chlorinated | UK | London | N5-4-2 | N5 | Not-disinfected | Netherlands | Amsterdam |
| UK5-3-4 | UK5 | Chlorinated | UK | London | N5-4-3 | N5 | Not-disinfected | Netherlands | Amsterdam |
| UK5-4-1 | UK5 | Chlorinated | UK | London | N5-4-4 | N5 | Not-disinfected | Netherlands | Amsterdam |
| UK5-4-2 | UK5 | Chlorinated | UK | London |  |  |  |  |  |
| UK5-4-3 | UK5 | Chlorinated | UK | London |  |  |  |  |  |
| UK5-4-4 | UK5 | Chlorinated | UK | London |  |  |  |  |  |
| UK6-1-3 | UK6 | Chlorinated | UK | London |  |  |  |  |  |
| UK6-1-4 | UK6 | Chlorinated | UK | London |  |  |  |  |  |
| UK6-2-1 | UK6 | Chlorinated | UK | London |  |  |  |  |  |
| UK6-2-2 | UK6 | Chlorinated | UK | London |  |  |  |  |  |
| UK6-2-3 | UK6 | Chlorinated | UK | London |  |  |  |  |  |
| UK6-2-4 | UK6 | Chlorinated | UK | London |  |  |  |  |  |
| UK6-3-1 | UK6 | Chlorinated | UK | London |  |  |  |  |  |
| UK6-3-2 | UK6 | Chlorinated | UK | London |  |  |  |  |  |
| UK6-3-3 | UK6 | Chlorinated | UK | London |  |  |  |  |  |
| UK6-3-4 | UK6 | Chlorinated | UK | London |  |  |  |  |  |
| UK7-1-1 | UK7 | Chlorinated | UK | London |  |  |  |  |  |
| UK7-1-2 | UK7 | Chlorinated | UK | London |  |  |  |  |  |
| UK7-1-3 | UK7 | Chlorinated | UK | London |  |  |  |  |  |
| UK7-1-4 | UK7 | Chlorinated | UK | London |  |  |  |  |  |
| UK7-2-1 | UK7 | Chlorinated | UK | London |  |  |  |  |  |
| UK7-2-2 | UK7 | Chlorinated | UK | London |  |  |  |  |  |
| UK7-2-3 | UK7 | Chlorinated | UK | London |  |  |  |  |  |
| UK7-2-4 | UK7 | Chlorinated | UK | London |  |  |  |  |  |

**Table S2**. Assembly quality of contigs assessed with QUAST

| Assembly | U1.final.contigs | U2.final.contigs | U3.final.contigs | U4.final.contigs | U5.final.contigs | U6.final.contigs | U7.final.contigs |
| --- | --- | --- | --- | --- | --- | --- | --- |
| # contigs (>= 0 bp) | 117893 | 36945 | 176113 | 124332 | 198908 | 37555 | 75445 |
| # contigs (>= 1000 bp) | 30579 | 11471 | 56773 | 41278 | 56405 | 12989 | 23591 |
| # contigs (>= 5000 bp) | 2996 | 1010 | 4763 | 4961 | 5363 | 901 | 1782 |
| # contigs (>= 10000 bp) | 1384 | 367 | 1852 | 2085 | 1986 | 375 | 817 |
| # contigs (>= 25000 bp) | 395 | 126 | 471 | 676 | 452 | 145 | 331 |
| # contigs (>= 50000 bp) | 109 | 62 | 165 | 288 | 110 | 63 | 119 |
| Total length (>= 0 bp) | 153374424 | 52826005 | 242311893 | 2.06E+08 | 253581364 | 54068173 | 105845896 |
| Total length (>= 1000 bp) | 95367604 | 35852307 | 161383965 | 1.5E+08 | 158963361 | 37242739 | 70803630 |
| Total length (>= 5000 bp) | 47223348 | 16632766 | 69740074 | 82650739 | 67210775 | 16046693 | 32519166 |
| Total length (>= 10000 bp) | 36104036 | 12239964 | 49800669 | 62999492 | 44274899 | 12527532 | 25980512 |
| Total length (>= 25000 bp) | 21230888 | 8726430 | 29199061 | 41906773 | 21697898 | 8969412 | 18515877 |
| Total length (>= 50000 bp) | 11387676 | 6510822 | 18586322 | 28295709 | 10223077 | 6079787 | 11131103 |
| # contigs | 117893 | 36945 | 176113 | 124332 | 198908 | 37555 | 75445 |
| Largest contig | 502373 | 214117 | 559589 | 350451 | 536732 | 303368 | 276298 |
| Total length | 153374424 | 52826005 | 242311893 | 2.06E+08 | 253581364 | 54068173 | 105845896 |
| GC (%) | 59.83 | 55.34 | 58.15 | 57.65 | 59.35 | 59.29 | 61.19 |
| N50 | 1524 | 1920 | 1662 | 2769 | 1510 | 1747 | 1723 |
| N75 | 740 | 818 | 815 | 934 | 743 | 860 | 816 |
| L50 | 15105 | 4428 | 24751 | 10541 | 29763 | 5059 | 9606 |
| L75 | 53762 | 15659 | 79414 | 45539 | 93054 | 16569 | 33146 |
| # N's per 100 kbp | 0 | 0 | 0 | 0 | 0 | 0 | 0 |
| Assembly | N1.final.contigs | N2.final.contigs | N3.final.contigs | N4.final.contigs | N5.final.contigs |  |  |
| # contigs (>= 0 bp) | 434589 | 276457 | 577829 | 146352 | 424277 |  |  |
| # contigs (>= 1000 bp) | 94209 | 56545 | 106549 | 28178 | 111943 |  |  |
| # contigs (>= 5000 bp) | 3749 | 2083 | 3816 | 1303 | 5874 |  |  |
| # contigs (>= 10000 bp) | 1166 | 597 | 1433 | 428 | 1794 |  |  |
| # contigs (>= 25000 bp) | 184 | 127 | 334 | 77 | 426 |  |  |
| # contigs (>= 50000 bp) | 45 | 40 | 73 | 25 | 111 |  |  |
| Total length (>= 0 bp) | 412194081 | 258002133 | 517809378 | 135117524 | 4.57E+08 |  |  |
| Total length (>= 1000 bp) | 187722440 | 113478822 | 208938915 | 57796982 | 2.47E+08 |  |  |
| Total length (>= 5000 bp) | 40695785 | 24086733 | 47376948 | 14484736 | 67279394 |  |  |
| Total length (>= 10000 bp) | 23302644 | 14313696 | 31282392 | 8538209 | 40029150 |  |  |
| Total length (>= 25000 bp) | 8887070 | 7436176 | 14484524 | 3475071 | 19860983 |  |  |
| Total length (>= 50000 bp) | 4274078 | 4400789 | 5630995 | 1818118 | 9288410 |  |  |
| # contigs | 434589 | 276457 | 577829 | 146352 | 424277 |  |  |
| Largest contig | 280666 | 483502 | 409981 | 141622 | 497436 |  |  |
| Total length | 412194081 | 258002133 | 517809378 | 135117524 | 4.57E+08 |  |  |
| GC (%) | 53.77 | 53.2 | 53.16 | 56.83 | 57.05 |  |  |
| N50 | 916 | 888 | 840 | 872 | 1092 |  |  |
| N75 | 638 | 631 | 619 | 623 | 694 |  |  |
| L50 | 113430 | 73056 | 161368 | 38665 | 94087 |  |  |
| L75 | 250905 | 161057 | 343927 | 85390 | 228523 |  |  |
| # N's per 100 kbp | 0 | 0 | 0 | 0 | 0 |  |  |

**Table S3** Quality of viral contigs assessed with QUAST

| Assembly | free viral contig | prophage contig |
| --- | --- | --- |
| # contigs (>= 1000 bp) | 13819 | 405 |
| # contigs (>= 5000 bp) | 4353 | 220 |
| # contigs (>= 10000 bp) | 1173 | 122 |
| # contigs (>= 25000 bp) | 187 | 46 |
| # contigs (>= 50000 bp) | 31 | 5 |
| Total length (>= 0 bp) | 74587267 | 4322207 |
| Total length (>= 1000 bp) | 74048606 | 4312334 |
| Total length (>= 5000 bp) | 43385599 | 3704740 |
| Total length (>= 10000 bp) | 22014705 | 3018505 |
| Total length (>= 25000 bp) | 7820905 | 1873247 |
| Total length (>= 50000 bp) | 2617992 | 381209 |
| # contigs | 14489 | 417 |
| Largest contig | 242045 | 116368 |
| Total length | 74577890 | 4322207 |
| GC (%) | 51.44 | 59.74 |
| N50 | 5913 | 19018 |
| N75 | 3789 | 8141 |
| L50 | 3229 | 60 |
| L75 | 7261 | 147 |
| # N's per 100 kbp | 0 | 0 |

**Table S4** Mantel Test of prokaryotic antiviral systems and water quality parameters

| Data1 | Data2 | rho | pvalue |
| --- | --- | --- | --- |
| antiviral system | Temp | 0.19497931529754 | 0.002 |
| antiviral system | pH | -0.0064327776864408 | 0.456 |
| antiviral system | Conductivity | 0.18002385140738 | 0.001 |
| antiviral system | DO | 0.097994565864112 | 0.052 |
| antiviral system | Chlorine | 0.29294625331141 | 0.001 |
| antiviral system | Phosphate | 0.23453274539007 | 0.001 |
| antiviral system | TOC | -0.14410059558500 | 1 |
| antiviral system | Ammonia | 0.23195104018466 | 0.001 |
| antiviral system | Nitrate | 0.1838538356386 | 0.005 |

**Table S5** 46 types of antiviral systems were identified

| Antiviral system name | Abundance in Chlorinated DWDS | Abundance in not-disinfected DWDS | Percentage of this system among all antiviral systems in Chlorinated DWDS (%) | Percentage of this system among all antiviral systems in not-disinfected DWDS (%) |
| --- | --- | --- | --- | --- |
| AVAST | 1.84E-05 | 3.75E-06 | 0.25 | 0.67 |
| Abi2 | 2.16E-05 | 8.87E-06 | 5.51 | 11.74 |
| AbiEiI | 4.86E-04 | 1.57E-04 | 0.03 | 0.12 |
| AbiH | 2.51E-06 | 1.54E-06 | 0.21 | 0.28 |
| BREX | 6.76E-04 | 1.79E-05 | 7.67 | 1.34 |
| BstA | 7.09E-07 | 6.33E-07 | 0.01 | 0.05 |
| CAS | 1.35E-03 | 2.01E-04 | 15.30 | 15.08 |
| CBASS | 4.85E-04 | 1.13E-04 | 5.51 | 8.48 |
| DISARM | 1.56E-05 | 8.06E-07 | 2.05 | 1.42 |
| DRT | 5.25E-06 | 5.16E-06 | 0.20 | 0.80 |
| DarTG | 1.81E-04 | 1.90E-05 | 0.05 | 0.16 |
| Dnd | 4.17E-06 | 1.70E-06 | 0.18 | 0.06 |
| DrB | 5.13E-05 | 3.66E-06 | 0.05 | 0.13 |
| Dsr | 8.87E-05 | 7.84E-06 | 0.58 | 0.27 |
| Gabija | 2.00E-04 | 2.46E-05 | 0.06 | 0.39 |
| Gao | 6.88E-05 | 1.73E-05 | 1.01 | 0.59 |
| GasderMIN | 8.28E-08 | 8.71E-07 | 2.27 | 1.84 |
| Hachiman | 1.00E-04 | 9.26E-06 | 0.78 | 1.30 |
| Kiwa | 2.43E-05 | 3.36E-06 | 0.00 | 0.07 |
| LamassB | 4.50E-06 | 1.19E-06 | 1.14 | 0.69 |
| Lit | 4.53E-06 | 4.29E-06 | 0.28 | 0.25 |
| NhI | 3.34E-06 | 6.52E-07 | 0.05 | 0.09 |
| NixI | 2.43E-07 | 1.46E-07 | 0.05 | 0.32 |
| PARIS | 2.17E-05 | 6.80E-06 | 0.04 | 0.05 |
| PrrC | 5.00E-05 | 1.78E-05 | 0.00 | 0.01 |
| RM | 4.49E-03 | 5.75E-04 | 0.25 | 0.51 |
| Retron | 5.17E-05 | 2.08E-05 | 0.57 | 1.34 |
| Rst | 1.58E-05 | 4.93E-06 | 0.59 | 1.56 |
| SeptB | 4.54E-05 | 1.50E-05 | 50.98 | 43.14 |
| ShedB | 2.26E-05 | 2.05E-05 | 0.18 | 0.37 |
| SspBCDE | 2.26E-05 | 1.25E-06 | 0.51 | 1.13 |
| Thoeris | 1.03E-05 | 6.98E-06 | 0.26 | 1.54 |
| Viperin | 1.35E-07 | 5.39E-07 | 0.26 | 0.09 |
| Wadjet | 1.24E-04 | 4.09E-05 | 0.12 | 0.52 |
| Zorya | 1.43E-04 | 6.71E-06 | 0.00 | 0.04 |
| dCTPdeaminase | 1.72E-05 | 1.07E-05 | 1.41 | 3.07 |
| dGTPase | 4.13E-06 | 2.14E-06 | 1.62 | 0.50 |

**Table S6** Mantel Test of viral contig and water quality parameters

| Data1 | Data2 | rho | pvalue | relation |
| --- | --- | --- | --- | --- |
| vOUT | Temp | 0.346346 | 0.001 | positive |
| vOUT | pH | 0.059964 | 0.022 | positive |
| vOUT | Conductivity | 0.380437 | 0.001 | positive |
| vOUT | DO | 0.157542 | 0.001 | positive |
| vOUT | Chlorine | 0.412148 | 0.001 | positive |
| vOUT | Phosphate | 0.407141 | 0.001 | positive |
| vOUT | TOC | 0.148435 | 0.001 | positive |
| vOUT | Ammonia | 0.136983 | 0.001 | positive |
| vOUT | Nitrate | 0.236701 | 0.001 | positive |
